# PsychiatryBench: a multi-task benchmark for LLMs in psychiatry

**DOI:** 10.1038/s41746-026-02582-w

**Published:** 2026-04-14

**Authors:** Aya E. Fouda, Abdelrahman A. Hassan, Radwa J. Hanafy, Mohammed E. Fouda

**Affiliations:** 1 Compumacy for Artificial Intelligence Solutions, Cairo, Egypt; 2 Department of Behavioural Health- Saint Elizabeths Hospital, Washington, DC USA

**Keywords:** Business and industry, Health care, Medical research, Psychology, Psychology

## Abstract

Large language models (LLMs) offer significant potential in enhancing psychiatric practice, from improving diagnostic accuracy to streamlining clinical documentation and therapeutic support. However, existing evaluation resources heavily rely on small clinical interview corpora, social media posts, or synthetic dialogs, which limit their clinical validity and fail to capture the full complexity of diagnostic reasoning. In this work, we introduce PsychiatryBench, a rigorously curated benchmark grounded exclusively in authoritative, expert-validated psychiatric textbooks and casebooks. PsychiatryBench comprises eleven distinct question-answering tasks ranging from diagnostic reasoning and treatment planning to longitudinal follow-up, management planning, clinical approach, sequential case analysis, and multiple-choice/extended matching formats totaling 5,188 expert-annotated items. We evaluate a diverse set of frontier LLMs (including Google Gemini, DeepSeek, Sonnet 4.5, and GPT 5) alongside leading open-source medical models such as MedGemma using both conventional metrics and an “LLM-as-judge” similarity scoring framework. Our results reveal substantial gaps in clinical consistency and safety, particularly in multi-turn follow-up and management tasks, underscoring the need for specialized model tuning and more robust evaluation paradigms. PsychiatryBench offers a modular, extensible platform for benchmarking and improving LLM performance in mental health applications.

## Introduction

Mental disorders impose a staggering global burden, accounting for 5.1% of worldwide disease burden in 2019, affecting over 280 million people with depression, and claiming 703,000 lives to suicide^1^. The economic toll is projected to reach $14 trillion in the United States alone between 2024 and 2040, encompassing direct medical costs, emergency interventions, productivity losses, and premature mortality^2^. These figures demand urgent action: scalable technologies that can improve diagnostic precision, expand care access, and accelerate treatment delivery are not merely aspirational; they represent critical infrastructure gaps in modern healthcare systems.

Large language models (LLMs), a subset of artificial intelligence (AI), have emerged as a potentially transformative technology in healthcare, with significant implications for psychiatric practice. Recent studies demonstrate their ability to detect depression and suicide risk from text, generate clinical documentation preferred by psychiatrists in blinded comparisons, and even express empathy at levels exceeding human physicians in written communication^3–5^. LLMs are increasingly integrated across psychiatric workflows^6,7^, with applications ranging from detecting mental health conditions and suicidal ideation using social-media-derived text to operationalizing ICD-11 diagnostic structures into transparent logic systems suitable for clinician inspection^8^. Beyond assessment, CBT-grounded conversational agents such as Woebot and Wysa offer scalable digital mental health support^9,10^. In primary care, LLM-generated responses have in some cases been perceived as more empathetic or higher quality than those produced by clinicians^11,12^. In administrative domains, LLMs show promise in automating clinical documentation, with therapists sometimes preferring LLM-generated notes in blinded evaluations^3,13^.

Despite such promise, LLM deployment in psychiatry remains constrained by critical safety concerns. Models frequently produce inaccurate or fabricated responses, so-called “hallucinations” which pose serious clinical risks in high-stakes psychiatric contexts^14^. Additional concerns include clinician over-reliance on opaque systems, bias amplification reflecting socioeconomic or geographic disparities, and uncertain reliability in crisis or risk-assessment contexts^15,16^. These challenges underscore the need for evaluation frameworks that can rigorously assess clinical accuracy, reasoning quality, and safety.

Efforts to evaluate LLMs in psychiatric settings have evolved alongside the availability of datasets, forming a central throughline in prior work. Early corpora such as DAIC-WOZ and E-DAIC^17^ provided structured interview transcripts and enabled developments such as the LMIQ framework, which maps unstructured patient narratives onto quantitative screening tools^11^. Subsequent research leveraged large-scale social media datasets, including Reddit mental health corpora and depression-detection collections^18^ to pretrain models such as MentalBERT and MentalRoBERTa, though these models inherited limitations from unverified self-report data and oversimplified symptom descriptions^8^.

More recent benchmarks attempt to incorporate conversational complexity. MentalChat16K introduced a blend of synthetic and anonymized clinical dialogs^19^, while automated annotation strategies such as SPAADE-DR^20^ generated multilabel diagnostic profiles that reflect real-world comorbidity. Although these developments broadened evaluation scope, reliance on synthetic data and LLM-as-annotator paradigms raises concerns about validity, bias propagation, and fidelity to clinical reasoning.

Parallel lines of research show LLMs achieving high balanced accuracy in classification tasks such as depression and PTSD detection^21^, supporting administrative workflows^22^, and exhibiting strong empathetic communication in controlled evaluations^23,24^. Yet many of these successes rely on datasets that lack clinical rigor^25,26^, and the majority of evaluation frameworks emphasize surface-level pattern matching rather than multi-step psychiatric reasoning^27–29^. Persistent issues including hallucinations^30^, inconsistent reasoning^31^, and limited real-world validation^32^ highlight the inadequacy of current approaches.

A core limitation across existing work is the lack of grounding in authoritative psychiatric resources that define clinical training and practice. Foundational materials such as *DSM-5 Clinical Cases* and *DSM-5-TR Clinical Cases*^33,34^, high-yield casebooks such as *100 Cases in Psychiatry*^35^ and *Case Files Psychiatry*^36^, specialized psychopharmacology references including *Stahl’s Essential Psychopharmacology*^37^, and geriatric psychiatry guides^38^ collectively represent decades of expert knowledge. Structured resources such as *DSM-5-TR Self-Exam Questions* provide standardized factual assessments essential for evaluating foundational psychiatric knowledge. These materials document diagnostic nuance, comorbidity patterns, treatment algorithms, population-specific considerations, and the layered reasoning processes intrinsic to psychiatric practice. Evaluating LLMs against these sources not synthetic or unverified corpora is essential for determining whether models can approach expert-level clinical reasoning.

Benchmark proposals such as MentalBench-10, which evaluates empathy, affective alignment, and advice quality using synthetic and user-generated dialogs^39^ and PsychBench which spans diagnosis, treatment, and communication with metrics such as TCAS, MMS, and RCR^40^ represent important steps but retain limitations. MentalBench-10 focuses primarily on supportive communication, while PsychBench’s dataset size and partial reliance on synthetic content constrain generalizability and clinical depth. These shortcomings emphasize the need for a benchmark that is both comprehensive and grounded exclusively in validated psychiatric materials.

To address this critical gap, we introduce PsychiatryBench, a novel evaluation framework constructed entirely from foundational psychiatric textbooks and expert-validated clinical cases. PsychiatryBench is designed with a single purpose: to provide a rigorous, standardized tool for comparative evaluation of LLM capabilities in psychiatry. Its objective is to assess whether LLMs can approximate the depth, complexity, and precision of real-world psychiatric reasoning and clinical decision-making.

We define high-stakes psychiatric applications as contexts where AI errors risk serious patient harm, including misdiagnosis leading to inappropriate or delayed treatment, failure to detect suicide risk, incorrect medication selection with adverse outcomes, or inappropriate crisis management. Such applications require evaluation methods that examine not only language fluency but also clinical accuracy, reasoning transparency, and appropriate acknowledgment of uncertainty.

**Scope:** PsychiatryBench focuses on adult psychiatry as a medical specialty. It targets clinical reasoning specific to psychiatric diagnosis and treatment and excludes psychotherapy, counseling techniques, and broader behavioral health domains. Child and adolescent psychiatry is excluded due to distinct developmental considerations necessitating a dedicated framework, while geriatric psychiatry is included due to its relevance to general adult practice. Although source cases occasionally reference medical mimics (e.g., thyroid disease, CNS pathology, delirium), these are not comprehensively represented. PsychiatryBench does not assess conversational coherence, turn-by-turn dialog management, or biopsychosocial formulation; instead, it prioritizes knowledge application and decision-making.

Throughout this work, the term *psychiatric reasoning* encompasses six complementary dimensions of competence, each associated with specific task types:

*Diagnostic reasoning:* Integrating history, presentation, and mental status findings to generate and differentiate diagnoses (Diagnosis, Classification tasks).

*Treatment decision-making:* Selecting evidence-based interventions tailored to diagnostic impression and patient characteristics (Treatment, Treatment Follow-Up tasks).

*Management planning:* Coordinating care, risk assessment, psychoeducation, and longitudinal follow-up (Management Plan tasks).

*Clinical approach:* Structuring evaluations, prioritizing hypotheses, and identifying appropriate investigations (Clinical Approach tasks).

*Foundational knowledge:* Demonstrating factual understanding of diagnostic criteria, psychopharmacologic mechanisms, and clinical principles (Mental QA, MCQ, EMI tasks).

*Sequential reasoning:* Adapting diagnostic and treatment recommendations over evolving clinical timepoints (Sequential QA tasks).

Our contribution comprises three interconnected elements:

- A clinically rigorous dataset curated from authoritative psychiatric casebooks, diagnostic manuals, and specialized clinical guides, ensuring grounding in validated expert knowledge rather than unverified or synthetic data.
- Eleven complex task types that rigorously evaluate whether LLMs are prepared for the intellectually demanding and ethically high-stakes reasoning required in psychiatric practice.
- A standardized evaluation resource for comparative LLM assessment in psychiatry. While insights may inform discussions about decision support or educational applications, PsychiatryBench’s primary purpose is rigorous model evaluation; regulatory or policy implications would require additional considerations outside the scope of this work.

Taken together, PsychiatryBench introduces a clinically grounded, multifaceted, and extensible evaluation paradigm designed to determine whether LLMs can approximate the depth, precision, and safety required for high-stakes psychiatric care, addressing longstanding gaps in prior benchmarks and providing a foundation for responsible integration of LLMs into psychiatric workflows.

## Results

**Fig. 1:**
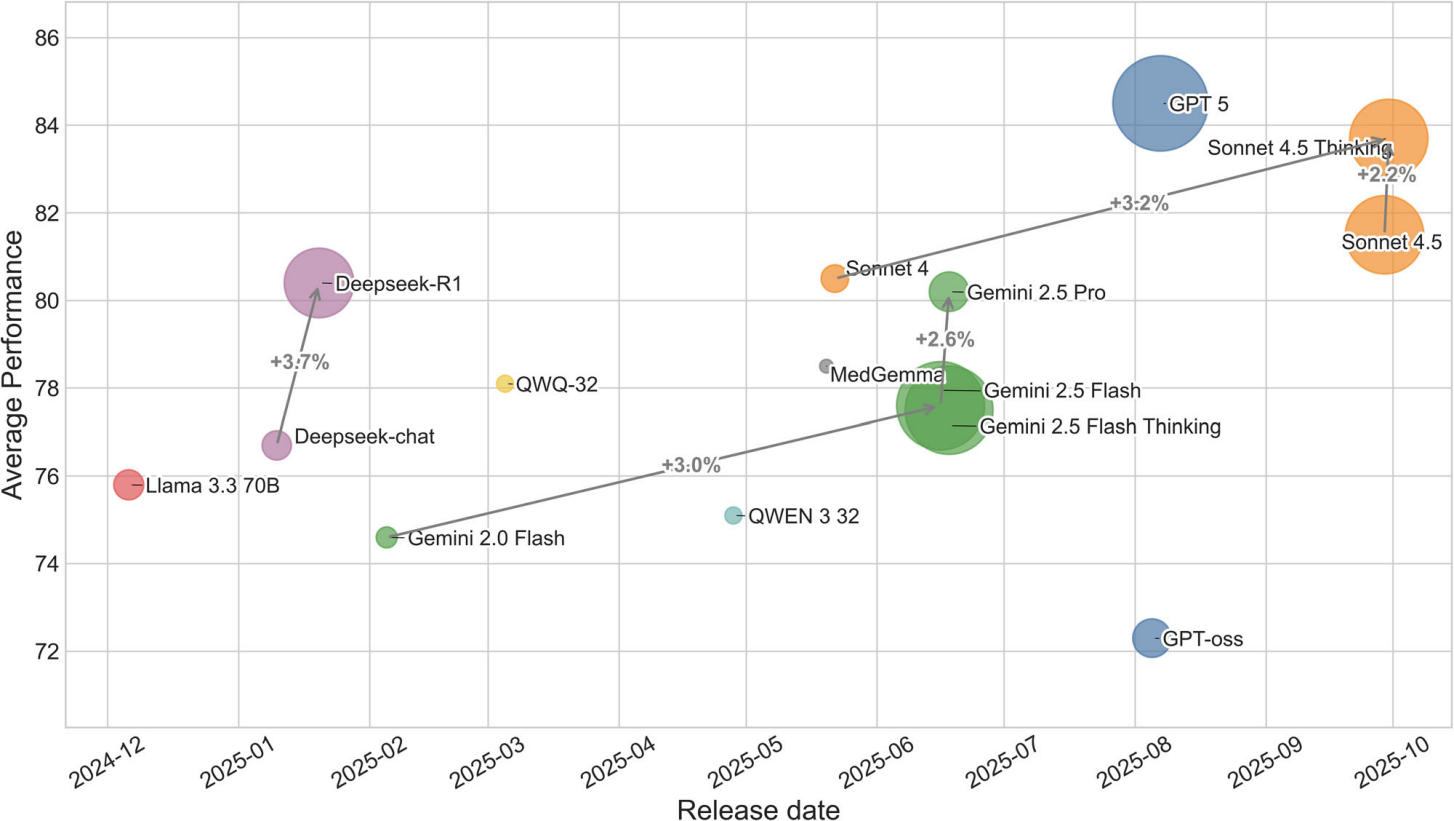
Each bubble shows a model, positioned by release date and average performance. Bubble size denotes parameter count, and color represents model family. Larger, newer models generally achieve higher performance.

This section presents a multi-faceted analysis of the performance of 15 leading LLMs on the PsychiatryBench benchmark. Our evaluation, summarized in Table 2, provides a granular view of each model’s capabilities across eleven distinct diagnostic reasoning tasks. Complementing this, Fig. 1 offers a macroscopic perspective, plotting each model’s average performance against its release date, with bubble size representing the model’s parameter count. This integrated approach allows for a thorough discussion of overarching performance trends, the impact of model scale, the value of domain-specific training, and the efficacy of different inference strategies.

### Performance of medical models

In this section, we evaluate the medical models submitted to establish a clinically meaningful baseline. Table 1 reports the performance of several specialized medical LLMs alongside multiple frontier models. II-Medical-8B attains (76.8%) on both the Management Plan and Mental QA, indicating well-balanced performance across tasks. OpenBioLLM achieves (75.1%) on the Management Plan and a higher (82.2%) on Mental QA. Med-Palm (v2) obtains similarly competitive results, with (78.2%) and (83.2%), respectively. In contrast, JSL-MedLlama performs substantially worse, scoring (61.0%) on Management Plan and (65%) on Mental QA, falling well below the 75% threshold and therefore being excluded from subsequent specialized analyses. Med-PaLM (v2) was also excluded due to deployment constraints that require formal Google approval for medical use cases, which prevented local fine-tuning and evaluation.

**Table 1:** Evaluation of medical and frontier LLMs on clinical diagnosis and follow-up tasks using benchmark datasets

Models	Management plan	Mental QA
II-Medical-8B	76.8	76.8
OpenBioLLM	75.1	82.2
MedGemma	$\underline{{\bf{81.7}}}$	$\underline{{\bf{87.1}}}$
Med-PaLM (v2)	78.2	83.2
JSL-MedLlama	61.0	65.0
Gemini 2.5 Flash	81.1	83.5
Gemini 2.5 Flash (T)	82.4	79.3
Deepseek-chat	83.5	82.7

The **underlined bold** values indicate the best score specifically among the listed medical models.

The underlined bold entries in Table 1 denote the best-performing medical models within this subset, rather than the best overall scores. This distinction is important because several general-purpose frontier LLMs evaluated elsewhere in the manuscript achieve markedly higher performance on PsychiatryBench, surpassing even the strongest medical models. Accordingly, the underline identifies the top results within the medical-model category only, not the global optimum across all models considered.

We further evaluated the medical language models using two complementary benchmark datasets, Management Plan and Mental QA, each targeting distinct aspects of clinical competence. Together, these datasets yield a balanced assessment of practical clinical decision-making and domain-specific psychiatric knowledge, providing a comprehensive characterization of medical LLM performance.

Among all medical LLMs examined, MedGemma attains the strongest overall results, with (81.7%) on Management Plan and (87.1%) on Mental QA. These clinical benchmarks also help contextualize the notably stronger performance of general-purpose frontier LLMs. For example, Deepseek-chat reaches (83.5%) on Management Plan, and Gemini 2.5 Flash (T) achieves (82.4%), both outperforming MedGemma. While the medical models exhibit strong biomedical knowledge and structured reasoning, the frontier LLMs demonstrate superior coherence, adaptability, and open-ended reasoning capabilities that are particularly valuable for psychiatric assessment. In our benchmarking, we chose MedGemma to be representative to medical models in the benchmarking against other SoTA LLMs.

### Overall performance landscape and a clear trajectory of improvement

The results unequivocally demonstrate a stratified performance landscape and a rapid, positive trajectory of advancement in LLM capabilities for diagnostic reasoning. As detailed in Table 2, a distinct top tier of models has emerged. GPT 5 Medium (T) stands out as the premier model with the highest average score of (84.5%), closely followed by Sonnet 4.5 (T) (83.7%). These models consistently deliver state-of-the-art performance, securing the best or second-best scores across the majority of tasks, particularly those involving complex clinical judgment like Diagnosis, Treatment, and management plan.

**Table 2:** Performance comparison of 15 selected LLMs across all PsychiatryBench tasks

Model	Diagnosis	Treatment	Treatment follow-Up	Classification		Management plan	Clinical approach	Mental QA	Sequential QA	MCQ	Exams	EMI	AVG.
				Categories	Specific								
DeepSeek -R1	84.2	79.9	78.7	0.72/54.0	0.50/44.0	84.4	90.0	88.0	89.0	83.2	79.2	85.8	80.4
DeepSeek -chat	82.7	81.9	79.4	0.63/31.0	0.41/23.0	83.5	90.1	82.7	88.9	75.8	71.4	79.8	76.7
Gemini 2.0 Flash	83.1	77.4	66.5	0.63/31.0	0.46/31.0	78.7	86.7	82.7	90.0	75.6	69.9	75.5	74.6
Gemini 2.5 Pro	86.0	80.5	86.5	0.60/26.0	0.42/31.0	84.1	89.2	87.0	96.9	81.7	80.4	87.9	80.2
Gemini 2.5 Flash	78.0	81.0	80.2	0.68/47.0	0.44/36.0	81.1	88.6	83.5	89.2	79.5	73.1	84.5	77.6
Gemini 2.5 Flash (T)	85.0	82.9	82.6	0.60/26.0	0.37/14.0	82.4	87.5	79.3	89.7	79.4	78.4	86.1	77.5
Llama 3.3 70B	83.6	82.3	75.0	0.68/46.0	0.47/34.0	82.1	89.7	84.2	81.4	77.1	66.5	72.2	75.8
QWQ-32	85.9	82.1	83.7	0.65/38.0	0.45/33.0	82.9	89.2	86.0	86.2	78.4	73.4	79.8	78.1
QWEN 3 32B	82.1	81.7	80.9	0.58/25.0	0.35/14.0	80.3	90.0	90.6	89.7	74.2	65.3	73.1	75.1
MedGemma	79.4	84.2	79.2	0.55/34.0	0.69/45.0	81.7	90.0	87.1	87.7	87.4	69.2	72.5	78.5
GPT-oss	70.8	67.8	64.5	0.68/53.0	0.46/39.0	72.2	73.5	86.3	93.4	74.6	72.2	78.4	72.3
GPT 5 Medium (T)	88.2	88.0	90.0	0.75/59.0	0.52/45.0	87.9	90.1	90.7	96.1	87.1	79.8	89.1	84.5
Sonnet 4	85.8	84.5	83.5	0.69/45.0	0.47/34.0	84.8	90.0	89.4	89.6	81.6	77.0	83.9	80.5
Sonnet 4.5	89.6	86.6	89.3	0.70/43.0	0.44/30.0	87.0	90.2	81.8	95.6	83.0	75.6	84.9	81.5
Sonnet 4.5 (T)	89.9	88.4	90.4	0.73/55.0	0.40/33.0	88.6	87.5	92.5	96.2	89.2	80.0	88.9	83.7

Classification tasks are evaluated using F1 Score and Subset Accuracy (F1/A), while other tasks are assessed based on the similarity between model responses and reference answers, as judged by an LLM evaluator. (T) denotes Thinking models. Green, blue, and red highlight the best, second-best, and lowest scores per column, respectively.

Conversely, a clear performance gap separates these leaders from earlier and smaller models. Gemini 2.0 Flash (74.6%) and GPT-oss (72.3%) anchor the lower end of the performance spectrum. Figure 1 visually articulates this evolutionary trend, revealing a strong positive correlation between model release date and average performance. For instance, average scores improve by over +8 points between Gemini 2.0 Flash (74.6%) and the Sonnet 4.5 series (83.5% -83.7%), and by more than +10 points relative to baselines such as GPT-oss (72.3%). The jump of +4.0 points from Sonnet 4 (80.5%) to GPT 5 Medium (T) (84.5%) in a matter of months underscores the accelerated pace of innovation. This progression suggests that continuous refinements in model architecture, training methodologies, and data curation are yielding substantial and measurable improvements in clinical applicability.

### Task-specific performance analysis: identifying strengths and persistent challenges

A horizontal analysis of Table 2 reveals consistent patterns in task difficulty across models. Modern LLMs demonstrate remarkable proficiency in tasks that require synthesizing contextual information and generating structured, long-form clinical reasoning. This is most evident in Sequential QA, where Sonnet 4.5 (T) achieved a near-perfect score of (96.2%). Similarly, strong performance in the Clinical Approach task topped at (90.2%) by Sonnet 4.5 shows that contemporary models are adept at constructing coherent diagnostic and management pathways from complex psychiatric vignettes.

Conversely, two task categories remain persistent challenges, exposing the limits of current LLM capabilities. First, the Classification of Specific Disorders task proved to be the most difficult across the benchmark, with even top-tier models like GPT 5 Medium (T) achieving only (0.52/45.0) F1-score/subset-accuracy. This reflects the inherent difficulty of multi-label classification in psychiatry, where overlapping symptoms and comorbidities blur categorical boundaries. Second, the Extended Matching Items (EMI) task, which demands discriminating among numerous clinically similar options, also showed broad performance variability. While models like GPT 5 Medium (T) scored (89.1%), Gemini 2.0 Flash recorded a much lower score of (75.5%) in this task. Separately, on the Exams task, Gemini 2.0 Flash had one of the lowest results at (69.9%), underscoring broad difficulties in structured assessment tasks. These findings highlight that while LLMs have mastered structured narrative reasoning, fine-grained clinical discrimination remains a frontier for further optimization.

### Specialized vs. generalist models: the case of MedGemma

A compelling narrative within our results is the performance of the domain-specialized model, MedGemma. Despite its moderate size, MedGemma achieves an impressive average score of (78.5%), placing it on par with large-scale generalist models like Gemini 2.5 Pro (80.2%) and DeepSeek-R1 (80.4%). This performance is not uniform; rather, it is concentrated in areas that directly benefit from its specialized training on biomedical and clinical texts.

As highlighted in Table 2, MedGemma’s distinct advantage is evident in its strong F1/Subset Accuracy scores in the highly granular Classification of Specific Disorders task (0.69/45.0), where it outperforms nearly all generalist counterparts. Furthermore, it achieved one of the highest results in the knowledge-intensive MCQ task (87.4%), reflecting superior factual retention and domain recall.

However, this specialization comes with a trade-off, as MedGemma was less competitive in broader, open-ended reasoning tasks such as Management Plan (81.7%) and Sequential QA (87.7%) compared to top-tier generalist models like Sonnet 4.5 (T) (96.2%) and GPT 5 Medium (T) (96.1%). This suggests a dichotomy where generalist models excel at fluid, multi-step reasoning and contextual integration, while specialized models remain superior in precision-driven, knowledge-based clinical classification and recall.

### The role of inference strategies: “Thinking” vs. standard models

Our study’s inclusion of “Thinking” variants for the Gemini 2.5 Flash and Sonnet 4.5 models reveals that the benefit of more deliberative, multi-step inference is highly architecture-dependent. For the Anthropic models, this strategy yielded a significant performance dividend. Sonnet 4.5 (T) not only surpassed its standard counterpart with an average score of (83.7%) versus (81.5%) but also achieved the highest performance across several of the most cognitively demanding tasks: Diagnosis (89.9%), Treatment (88.4%), Treatment Follow-Up (90.4%), and Mental QA (92.5%). This indicates that for the Sonnet architecture, the “Thinking” mode effectively activates deeper and more structured clinical reasoning pathways.

In contrast, this advantage was not evident for the Google Gemini models. The average performance of Gemini 2.5 Flash (T) (77.5%) was negligibly lower than that of its standard counterpart, Gemini 2.5 Flash (77.6%). Moreover, the ’Thinking’ variant’s performance was mixed: it underperformed in Mental QA (79.3%) compared to the standard model’s (83.5%), but it scored higher in Diagnosis (85.0%) versus the standard model’s (78.0%), indicating an inconsistent benefit from the additional inference step.

### The impact of model scale and release date

The scatter plot in Fig. 1 provides critical insights into the relationship between model size (parameter count), recency, and overall performance. A clear upward trend emerges: larger models depicted by larger bubbles tend to cluster in the higher performance range. The leading results achieved by Sonnet 4.5 (T) and GPT 5 Medium (T), with average scores of (83.7%) and (84.5%) respectively, exemplify how scale combined with architectural refinement drives superior clinical reasoning outcomes.

However, size alone does not guarantee success. The performance of DeepSeek-R1, a large-scale model with an average score of (80.4%), remains strong yet is surpassed by more recent and structurally optimized models like Sonnet 4.5 (T) and GPT 5 Medium (T). This pattern underscores that architectural innovation, training corpus diversity, and post-alignment strategies can yield greater performance gains than mere parameter expansion.

The overall trajectory visible in Fig. 1 confirms this trend: newer architectures are consistently leveraging scale more effectively, converting size into meaningful improvements in reasoning accuracy, clinical adaptability, and task generalization.

### Model family performance

Our analysis reveals distinct performance signatures across model families, reflecting different architectural priorities and the effectiveness of their training. The frontier generalist models from Anthropic and OpenAI set the benchmark, with Sonnet 4.5 (T) (83.7%) and GPT-5 Medium (T) (84.5%) demonstrating state-of-the-art capabilities driven by recent architectural innovations.

In contrast, other families highlight key limitations. The Gemini models, while strong with Gemini 2.5 Pro at 80.2%, show that advanced inference modes are not universally beneficial; its “Thinking” variants yielded inconsistent gains, unlike the significant boosts seen in the Sonnet series. Similarly, DeepSeek-R1 (80.4%) illustrates that massive scale alone does not guarantee leadership, as it is surpassed by more structurally optimized models.

Finally, specialized medical models like MedGemma (78.5%) confirm a classic trade-off: their domain-specific pretraining provides a measurable edge in knowledge-intensive tasks but limits the cognitive flexibility needed for nuanced psychiatric reasoning. These patterns confirm that the frontier of performance is now defined more by sophisticated architectural design and alignment than by raw parameter count alone.

### Cross-task consistency and stability analysis

Beyond evaluating raw performance on individual tasks, an important dimension of Model reliability is the degree to which (LLMs) maintain stable behavior across the diverse task categories within PsychiatryBench. Given that the benchmark spans eleven clinically heterogeneous tasks ranging from structured classification to open-ended sequential reasoning cross-task consistency offers a deeper view of each model’s robustness, generalizability, and susceptibility to task-specific variability.

An inspection of the scores in Table 2 reveals substantial differences in cross-task stability among models. General-purpose frontier models such as Sonnet 4.5 (T) and GPT 5 Medium (T) demonstrate the highest degree of uniformity, maintaining strong performance across both highly structured tasks (e.g., classification of specific disorders) and open-ended reasoning tasks (e.g., sequential QA and clinical approach). This stability reflects the broad generalization capabilities of recent frontier models, supported by large-scale heterogeneous training data and refined inference strategies that preserve coherence across task formats. These models exhibit narrow performance fluctuations between their strongest and weakest tasks, indicating that their reasoning processes remain stable regardless of prompt structure or clinical domain.

In contrast, specialized medical models such as MedGemmathe and Med_Palmyra display a more uneven performance profile. Their strengths are concentrated in knowledge-intensive tasks such as mental QA and the classification of specific disorders, where domain-specific pretraining yields clear advantages. However, these models are noticeably less consistent on tasks requiring multi-step reasoning, contextual linking, or flexible narrative generation, such as sequential QA and clinical approach. The resulting performance gaps highlight a specialization-versus-generalization trade-off: while medical models excel when tasks directly match their training distribution, they are more sensitive to variations in clinical reasoning format or task structure.

Lower-performing models, exemplified by JSL_MedLlama, exhibit the widest instability across tasks. Large drops in performance between knowledge tasks and contextual reasoning tasks suggest that these models struggle to maintain coherent reasoning pipelines when task demands shift. Such volatility reduces their suitability for clinical applications where stability across problem types is a prerequisite for reliability.

Overall, the cross-task analysis underscores an important conclusion: the strongest models are not those that achieve isolated peaks on specific tasks, but those that maintain consistent performance across the full spectrum of psychiatric reasoning challenges. Frontier generalist models achieve the highest stability, while specialized models provide strong but localized competence. This distinction reinforces the need for multifaceted evaluation frameworks such as PsychiatryBench, which are capable of exposing not just peak performance but also the breadth and reliability of clinical reasoning capabilities.

### Extended matching item (EMI): full vs. separated evaluation

Based on the overall EMI-Full scores in Table 2, we restrict our format comparison to the five best-performing models on the EMI tasks: DeepSeek-R1, Gemini 2.5 Pro, Gemini 2.5 Flash (T), Sonnet 4.5 (T), and GPT-5. For these models, we compare performance on the original extended-set format (EMI-Full) and a separated evaluation in which each subquestion is scored independently. To ensure a fair item-by-item comparison, all metrics are computed on the overlapping subset of *N* = 144 EMI sets (*T* = 514 subquestions) that are structurally compatible across formats, with responses case-normalized (e.g., “a”/“A”) and scored under the same Answer Integrity Rule. A detailed description of this overlapping subset and the scoring procedure is provided in the Supplementary Information (see section “Extended matching item and separated extended matching item”).

Across this overlapping subset, the full-format results confirm that the selected models are already strong on EMI-Full. On the full 277-set benchmark, average PCS lies in a narrow band (approximately (85.8%–89.1)%), and when restricted to the 144 overlapping sets, full-format PCS remains high with a similar ordering: DeepSeek-R1 is clearly weaker at (72.0%), whereas Gemini 2.5 Flash (T) and Sonnet 4.5 (T) attain mid-70s to mid-80s PCS, and Gemini 2.5 Pro and GPT-5 reach (87.5%) and (87.2%), respectively. Gemini 2.5 Pro thus provides the strongest full-format performance on the overlapping subset, closely followed by GPT-5 and Sonnet 4.5 (T). Under the Separated evaluation, subquestion-level accuracy typically falls in the low-to-high (80%) range, with DeepSeek-R1 improving from (72.0%) PCS (full) to (83.6%) accuracy (separated), and Gemini 2.5 Flash (T) increasing from (75.7%) PCS to (88.1%) accuracy. In contrast, Gemini 2.5 Pro shifts only slightly from 87.5% PCS to (86.0%) accuracy, and GPT-5 from (87.2% to 87.8%), indicating that these models are already near their ceiling in the full format. Overall, Gemini 2.5 Pro achieves the highest full-format PCS on the overlapping subset, while Gemini 2.5 Flash (T) attains the highest separated accuracy (88.1%) and separated PCS (89.0%).

The consistency metrics summarized in the Supplementary Information (see section “Extended matching item and separated extended matching item”) provide additional insight into how stable each model’s behavior is across formats. Overall Consistency is highest for Gemini 2.5 Pro (95.7%) and GPT-5 (93.8%), with correspondingly low format divergence values of 0.043 and 0.062, indicating that these models change their correctness status between formats on only about 4–6% of overlapping subquestions. Gemini 2.5 Flash (T) and Sonnet 4.5 (T) achieve similarly strong accuracies but show somewhat higher divergence (around 0.13 and 0.20), suggesting more item-level sensitivity to format changes, while DeepSeek-R1 exhibits the lowest consistency (78.3%) and highest divergence (0.217) among the five models. Taken together, these score patterns indicate that the top EMI models identified in Table 2 generalize well across full-set and separated-subquestion formats, with Gemini 2.5 Pro and GPT-5 appearing particularly robust, and Gemini 2.5 Flash (T) benefiting the most from decomposing Extended Matching Items into separated subquestions.

## Discussion

The performance of contemporary LLMs on PsychiatryBench suggests that psychiatric reasoning is no longer at the edge of what these systems can do. A small group of recent frontier models, including GPT-5 Medium (T) (84.5%) and Sonnet 4.5 (T) (83.7%), form a clear top tier that maintains high accuracy across a diverse set of diagnostic and management tasks. Rather than indicating a Stagnation, the trend across model generations points to steady gains in psychiatric competence, driven primarily by architectural refinement and alignment strategies rather than parameter count alone. This temporal progression is clearly visible in Fig. 3, where average performance increases by over 10 percentage points across successive model generations. This trajectory mirrors broader developments in medical AI, but here it is achieved without explicit hand-crafted rules or specialty-specific expert systems.

A central conceptual finding is what we term the generalist-specialist paradox. Domain-specialized medical models, such as MedGemma and Med-Palm (v2), were expected to dominate on psychiatry-focused tasks given their training on biomedical and clinical corpora. Instead, they were consistently outperformed by general-purpose frontier models on complex reasoning tasks such as longitudinal follow-up, integrated management planning, and multi-step case synthesis. Specialized models retain a clear advantage in knowledge-intensive settings such as structured exams (MedGemma: 87.4%) or fine-grained disorder classification (F1: 0.69) where dense factual recall is paramount. However, psychiatric practice requires more than accurate retrieval: it depends on abstraction, contextual integration, and the ability to revise hypotheses as new information emerges. Our results therefore suggest that the most promising path for clinical AI may be to align powerful generalist models to psychiatric norms, rather than to build ever more narrowly trained specialty models.

Our evaluation of deliberative ("Thinking”) inference modes offers further insight into how these systems approximate clinical cognition. For some architectures, enabling explicit multi-step reasoning improves performance on the most demanding tasks and produces outputs that resemble careful differential diagnosis rather than rapid pattern matching. For others, the same strategy yields little benefit or even small regressions, indicating that simply “forcing” a chain-of-thought does not guarantee better clinical decisions. These findings highlight two requirements for psychiatric AI: first, models must possess latent reasoning capability; second, they must deploy it selectively and efficiently. This motivates the notion of clinical metacognition in AI systems knowing when additional deliberation is warranted and when simpler heuristics suffice.

Despite strong average scores, our analyses underscore reliability as a key barrier to safe deployment. Frontier models exhibit relatively stable behavior across very different task formats, whereas lower-tier and specialized models can oscillate between high performance on structured exams and serious failures on open-ended clinical prompts. Such volatility is particularly concerning in psychiatry, where an apparently competent model might provide accurate diagnostic labels yet hallucinate unsafe management plans or miss salient risk factors. Moreover, all models, including the strongest frontier systems, struggled with multi-label classification of specific disorders, with subset accuracy reaching only 45% even for GPT-5 Medium (T), and several showed sensitivity to modest changes in the Extended Matching Item (EMI) format. These weaknesses echo long-standing challenges in human psychiatry, including imperfect diagnostic agreement and the importance of subtle wording in assessments, but they also reveal areas where naïve deployment of LLMs could amplify misclassification or miscommunication.

From a clinical perspective, these patterns have several implications. First, benchmark performance cannot be interpreted as a uniform guarantee of safety: the same model may behave very differently on neighboring tasks that share content but differ in structure or required reasoning depth. Second, psychiatry-specific benchmarks need to probe not only correctness of final answers but also stability across formats, consistency over time, and the model’s handling of ambiguity, comorbidity, and partial information. Finally, our results argue for a cautious role for LLMs in psychiatric practice today, supporting education, documentation, or preliminary formulation rather than unsupervised decision-making in high-stakes scenarios such as crisis triage or medication initiation.

Our study has several limitations that shape the interpretation of these results and point to future work. Data sources and clinical representativeness. PsychiatryBench is intentionally grounded in authoritative textbooks and expert-validated casebooks. This design choice maximizes diagnostic precision and alignment with established standards, but it under-represents the noise, incomplete histories, and shifting narratives that characterize real-world clinical encounters. Classic teaching cases may overemphasize diagnostically “clean” or pedagogically rich scenarios and under-sample routine presentations or acute emergencies (for example, active suicidality, delirium, or mania requiring involuntary hospitalization). As a result, performance on PsychiatryBench may overestimate model reliability in everyday or crisis settings.Medical mimics (thyroid disorders, CNS conditions, delirium) are mentioned but not systematically included, restricting the evaluation of thorough differential diagnosis reasoning. Subsequent versions will emphasize the organized inclusion of these conditions. Additionally, the authoritative psychiatric textbooks that were utilized to develop PsychiatryBench are accessible to the public and are probably included in the pre-training data of the assessed models. This could lead to an artificial enhancement of performance through memorization instead of true reasoning. Although paraphrasing all items would undermine clinical authenticity, this risk of contamination should be taken into account when analyzing results. Future research should investigate methods for detecting contamination or create evaluation sets using unpublished clinical materials.

Scope of psychiatric practice. The benchmark focuses on adult psychiatry and incorporates some geriatric presentations, but it does not systematically address child and adolescent psychiatry, substance-use disorders in complex social environments, or cross-cultural variations in symptom expression and help-seeking. Nor does it cover the psychotherapy process, narrative formulation, or long-term therapeutic alliance. Furthermore, while recent studies indicate that LLMs may exhibit higher perceived empathy or documentation preference compared to physicians, we emphasize that these findings predominantly originate from primary care and non-psychiatric settings. Their transferability to psychiatry, where the therapeutic alliance is structurally distinct and documentation requires capturing subtle phenomenology, remains an open question not addressed by this benchmark. These omissions are deliberate, reflecting the goal of a focused, tractable first benchmark, but they limit the generalizability of our findings to the full breadth of psychiatric practice. Child and adolescent psychiatry was intentionally left out, even though many psychiatric disorders begin at an early age. This subspecialty demands unique developmental frameworks and will be covered in upcoming specific benchmarks. Furthermore, PsychiatryBench mainly depends on Western psychiatric resources (DSM-5-TR, American and European textbooks), which may not adequately reflect cultural differences in symptom display, help-seeking behaviors, or diagnostic methods. This signifies a present restriction that limits generalizability among various populations. Subsequent editions will include non-Western resources and culturally tailored diagnostic models

Evaluation methodology. Our primary metrics combine conventional scoring (for classification and multiple-choice items) with an LLM-as-judge framework for open-ended responses. While this approach is increasingly common and allows scalable evaluation of complex outputs, it introduces dependencies on the evaluator model’s biases, calibration, and sensitivity to superficial stylistic differences. Although we used standardized prompts and similarity-based rubrics, human experts would still be required to validate edge cases, particularly where safety concerns or subtle ethical issues are implicated. In addition, our evaluation is static: models interact with single prompts rather than longitudinal conversations or shared electronic health records, and we do not assess how clinicians might adapt or override model suggestions in practice. A significant limitation is the circularity inherent in using an LLM (Llama 3.3 70B) to evaluate other LLMs particularly as this same model was included among those evaluated. While grounding judgments in expert-validated reference answers and systematic judge selection mitigates this issue, it does not eliminate the circular dependency. Independent validation by panels of human psychiatrists remains essential for definitive benchmark validation.

System and deployment context. Finally, PsychiatryBench evaluates models in isolation. It does not capture the full socio-technical context of deployment, including user interfaces, guardrails, institutional policies, or multidisciplinary team oversight. Real-world safety will depend not only on model competence but also on workflow integration, documentation practices, audit mechanisms, and clear delineation of responsibility between clinicians and AI systems. PsychiatryBench fails to evaluate risks tied to patient-interactive situations, such as the reinforcement of delusions, improper management of acute suicidality, anthropomorphism, or sycophantic actions. These essential safety aspects necessitate specific assessment frameworks prior to clinical implementation.

Taken together, these limitations emphasize that PsychiatryBench should be viewed as a foundational evaluation resource, not a standalone certification of clinical readiness. Future work should extend the benchmark to include real-world clinical notes and dialogues, additional subspecialties and cultural contexts, multimodal inputs (such as brief mental-status videos or imaging reports), and explicit safety stress tests focused on crisis management and ethical boundary maintenance. Co-design with psychiatrists, patients, ethicists, and regulators will be essential to ensure that new tasks reflect genuine clinical priorities rather than purely technical benchmarks. Subsequent versions will systematically evaluate biopsychosocial formulations, functional impact assessments, and collaborative systems to more thoroughly represent multidisciplinary psychiatric practice.

Benchmark maintenance and sustainability: PsychiatryBench will be maintained and periodically updated by our team in collaboration with licensed psychiatrists to ensure continued clinical validity as diagnostic criteria, treatment guidelines, and AI capabilities evolve. Updates will address emerging psychiatric conditions, revised diagnostic criteria (e.g., DSM revisions), and newly identified model failure modes.

Nevertheless, our results show that state-of-the-art LLMs are beginning to approximate aspects of expert psychiatric reasoning while still exhibiting systematic vulnerabilities. PsychiatryBench helps to make these strengths and weaknesses visible by integrating diverse task types, probing both knowledge and reasoning, and highlighting where generalist and specialist models diverge. By grounding evaluation in authoritative clinical sources and emphasizing reliability and safety, this benchmark provides a structured basis for developing, aligning, and monitoring AI systems intended for mental healthcare, and for guiding the cautious, accountable integration of such systems into psychiatric practice.

The PsychiatryBench dataset was developed using content manually curated from publicly available, expert-authored psychiatric textbooks and casebooks. All material was selected and annotated by licensed psychiatrists with clinical backgrounds to support research in safe and effective psychiatric reasoning by AI models. Critically, no personal, identifiable, or patient-derived data were used in the construction of this dataset. The benchmark does not contain clinical notes, transcripts, or sensitive medical information from real individuals. All case vignettes, clinical scenarios, and diagnostic examples are drawn exclusively from published educational materials designed for training and assessment purposes in psychiatry.

This research adheres to ethical guidelines regarding the use of educational content for research purposes. The dataset is intended solely for non-commercial research, academic, and educational uses that aim to improve the safety, reliability, and clinical alignment of language models in mental health applications. All references to psychiatric diagnoses, treatments, and case scenarios are derived from simulated or publicly available educational materials, not real patient interactions. The benchmark’s design prioritizes patient privacy, professional standards, and responsible AI development.

Content in PsychiatryBench is derived from widely used psychiatric educational resources (including DSM-5-TR Clinical Cases, Stahl’s Essential Psychopharmacology, and 100 Cases in Psychiatry) under the principle of fair use for:

- Transformative use: Material has been substantially restructured into a novel benchmark format for evaluating AI systems, fundamentally changing its purpose from clinical education to computational assessment.
- Non-commercial research: The dataset is used exclusively for research and academic purposes without commercial exploitation.
- Limited scope: Only selected, representative samples were extracted not entire works and the benchmark does not substitute for or compete with the original textbooks.

Users are responsible for ensuring compliance with local copyright laws. Those intending to redistribute substantial portions, incorporate the dataset into commercial products, or use it beyond academic research should seek appropriate permissions from original copyright holders.

By using PsychiatryBench, researchers commit to:

- Citing original source materials appropriately
- Using the benchmark exclusively for research that prioritizes patient safety
- Engaging with psychiatric and AI ethics communities to maintain alignment with professional standards

This work is grounded in principles of responsible AI development, intellectual property respect, and patient-centered care.

## Methods

In this section, we present the comprehensive methodological framework used to develop and evaluate PsychiatryBench. We begin by detailing the curation of the dataset from authoritative psychiatric sources and the design of eleven distinct clinical task types. We then describe the experimental setup, including the selection of both general and domain-specific LLMs and the implementation of standardized prompting strategies. Finally, we outline the evaluation protocols, defining the metrics and the LLM-as-a-judge mechanism employed to assess clinical reasoning and decision-making capabilities.

### Dataset curation and task design

The dataset created in this study was manually curated to address the unique challenges of psychiatric reasoning, with a focus on a diverse range of clinically relevant tasks. Sourced from authoritative psychiatry textbooks and expert-validated clinical resources, the dataset comprises natural language questions paired with expert-formulated answers. These questions are grounded in real-world psychiatric scenarios. They are designed to evaluate the reasoning, decision-making, and knowledge application abilities of LLMs in both clinical and educational contexts. It is important to clarify the benchmark’s intended clinical setting and acuity level. PsychiatryBench is primarily designed as a research and pedagogical benchmark to support model development and the systematic evaluation of psychiatric reasoning tasks. As such, its current focus is on adult outpatient psychiatric reasoning and not severely acute clinical contexts.

This emphasis on outpatient scenarios, where structured diagnostic and treatment reasoning can be most effectively assessed, means that the benchmark does not comprehensively represent high-acuity, emergency, or inpatient psychiatry scenarios (such as acute mania requiring admission, delirium, or imminent suicidality). We acknowledge that these contexts represent distinct and critical aspects of clinical care. This focus is a deliberate scoping choice, and the exclusion of these high-acuity presentations is further discussed as a limitation in the Discussion section.

**Fig. 2:**
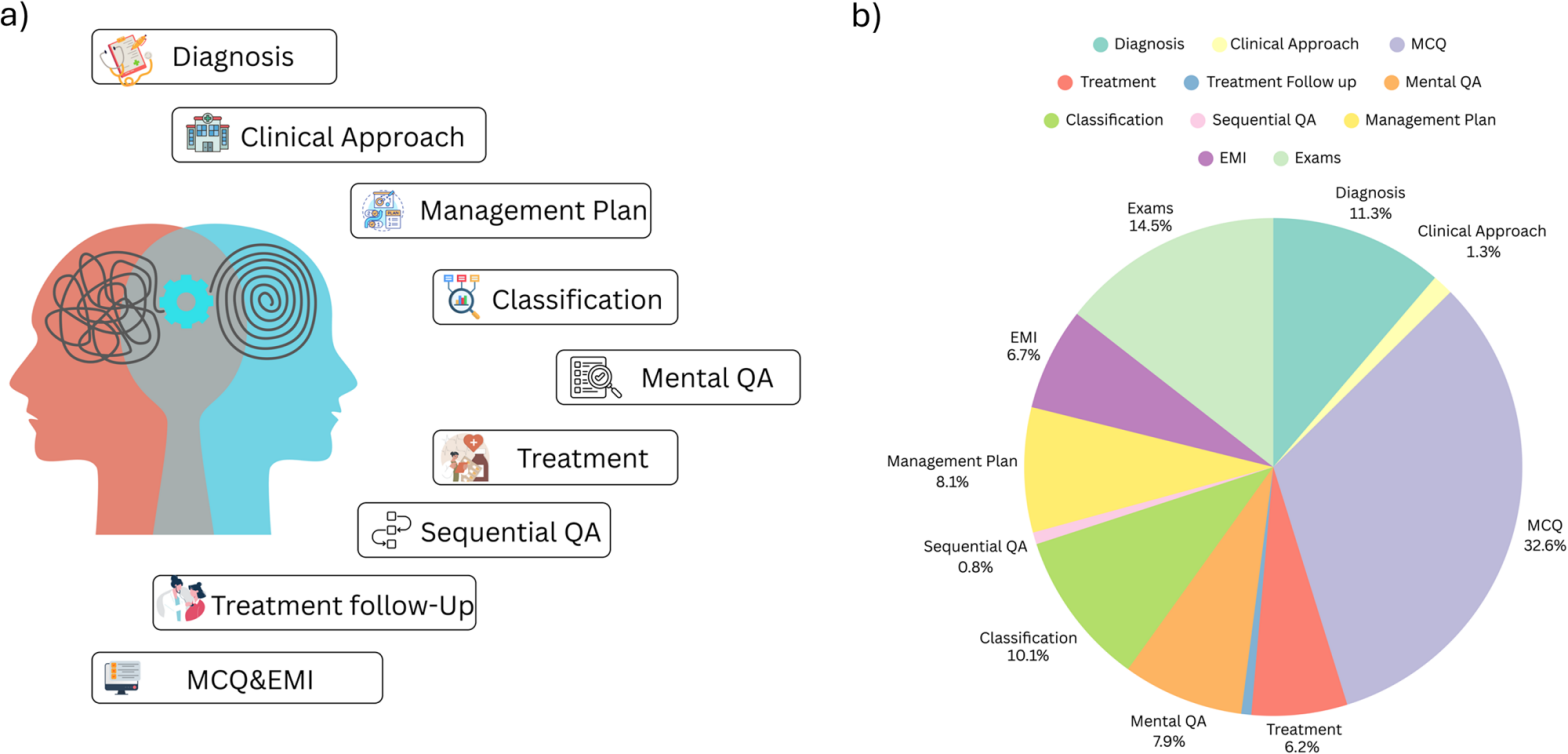
PsychiatryBench dataset composition and task distribution. **a** Overview of the full dataset development pipeline, illustrating the manual extraction and filtering steps used to construct Psychiatry Bench from authoritative psychiatric textbooks and casebooks. **b** Structure and percentage distribution of the selected evaluation subset (S-dataset) across eleven clinical task types, including Diagnosis (11.3%), MCQ (32.6%), Exams (14.5%), Classification (10.1%), Management Plan (8.1%), Mental QA (7.9%), EMI (6.7%), Treatment (6.2%), Clinical Approach (1.3%), and Sequential QA (0.8%).

Figure 2 provides a visual summary of the overall composition of PsychiatryBench across clinical task categories. Table 3 details the psychiatry textbooks and case-based resources used to construct the benchmark, indicating which clinical task types are covered by each source. Complementing this source-level overview, Table 4 outlines the quantitative distribution of samples across task types. Together, these figures and tables provide a comprehensive view of the dataset’s provenance, structural composition, and task diversity used in model evaluation.

**Table 3:** A summary of the psychiatry textbooks and case-based resources used for the dataset, showing the specific tasks covered by each source

Books	Diagnoses	Treatment	Treatment follow-Up	Classification	Management plan	Clinical approach	Mental QA	Sequential QA	MCQs
Geriatric psychiatry^38^	*✓*	*✓*			*✓*				*✓*
Case Files Psychiatry^36^	*✓*	*✓*		*✓*	*✓*	*✓*	*✓*		*✓*
Clinical Psychology Casebook^43^				*✓*					
DSM-5 Clinical Cases^33^	*✓*	*✓*	*✓*						*✓*
DSM-5-TR Self-Exam Questions^44^									*✓*
100 Cases in Psychiatry^35^	*✓*	*✓*		*✓*	*✓*		*✓*		
Core Clinical Cases^45^	*✓*			*✓*				*✓*	
Casebook in Abnormal Psychology^46^			*✓*	*✓*					
Revision MCQ and EMI^47^									*✓*
Psychiatry case vignettes^48^	*✓*			*✓*	*✓*		*✓*		
Clinical Cases in Psychiatry^49^			*✓*	*✓*		*✓*			*✓*
Stahl’s Psychopharmacology Cases^50^			*✓*						
Stahl’s Essential Cases^51^	*✓*			*✓*					*✓*

A check mark (*✓*) indicates coverage of a task.

**Table 4:** Distribution of available datasets categorized by clinical task type

Clinical task	Number of samples
Diagnoses	467
Treatment	258
Treatment follow-up	27
Classification	418
Management plan	337
Clinical approach	56
Mental QA	326
Sequential question answering	32
Multiple choice questions	1353
Extended matching items	277
Extended matching items separated	1037
Exams	600
Total	5188

The manual collection process ensured that the dataset maintains high-quality, medically accurate content, aligning with standardized diagnostic frameworks such as the DSM-5 and ICD-10, as well as clinical practice guidelines from organizations like the National Institute for Health and Care Excellence (NICE) and the American Psychiatric Association (APA).

#### PsychiatryBench development pipeline

**Fig. 3:**
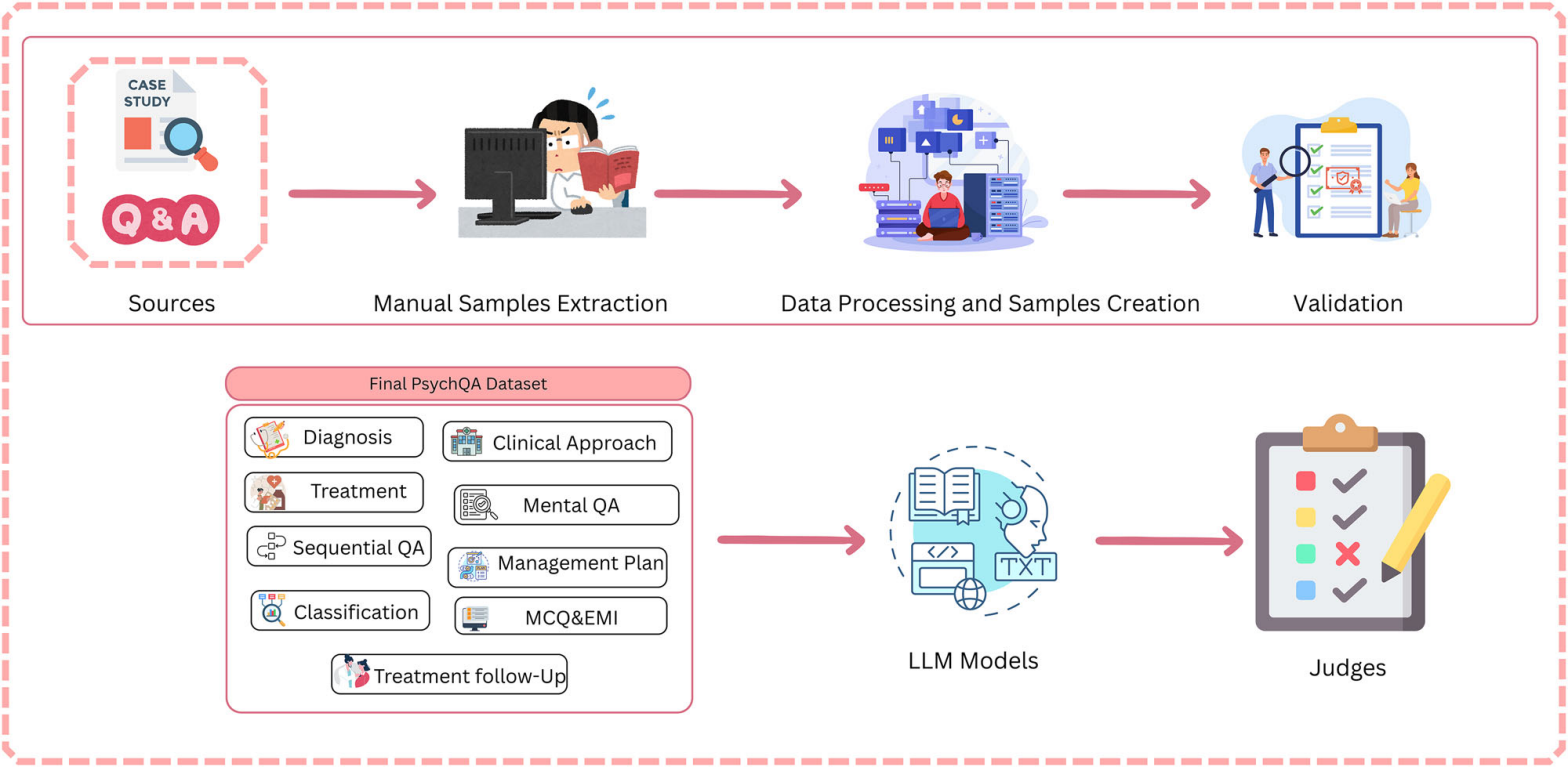
Workflow for the PsychiatryBench study: manual extraction and processing of clinical QA samples followed by LLM evaluation across eleven task types.

Our methodology, shown in Fig. 3, begins with systematic manual extraction of content from standard evidence-based psychiatry textbooks. licensed psychiatrist thoroughly review and select clinical material with high educational and diagnostic value, vibrant case studies, descriptive patient scenarios, and disorder-specific treatment information. From this material, a diverse set of question-answer (QA) pairs is constructed to represent real-world diagnostic reasoning. The QA items are crafted across eleven core task types: Diagnosis, Treatment, Mental QA, Sequential QA, Management plan, Treatment Follow-up, Clinical Approach, Classification, Multiple Choice Questions (MCQ), exams, and Extended Matching Items (EMI).

Following construction, the QA items are passed through a rigorous filtering process designed to ensure clarity, clinical validity, and educational utility. Ambiguous, overly simple, or redundant items are removed, and the remaining questions are manually reviewed for content balance and diagnostic diversity. These filtered QA items form the foundation of the PsychiatryBench dataset. In this phase, questions are sorted by task type and formatted consistently, allowing for structured downstream evaluation. A comprehensive data cleaning step is performed next, standardizing answer styles, resolving typographic issues, and ensuring uniform metadata. The result is a refined, high-quality QA dataset that tests not only factual recall but also diagnostic reasoning, treatment planning, and sequential clinical decision-making in psychiatry.

To evaluate the performance of LLMs, we apply each model to the finalized PsychiatryBench dataset. Each model is prompted to generate answers across all eleven selected task types. Importantly, in addition to serving as QA generators, LLMs are also used as evaluators or judges, scoring the generated responses based on accuracy, completeness, and clinical relevance. This dual use of LLMs helps automate and scale the evaluation process while maintaining consistency across judgments. A structured rubric is applied to produce a final score for each model, capturing performance across various clinical dimensions. This evaluation framework enables a robust and scalable benchmark for assessing how well LLMs understand, reason through, and respond to complex psychiatric questions, grounded in authentic medical education content.

Psychiatry Bench evaluates these dimensions across its eleven task types. We acknowledge that some of our source texts, such as DSM-5-TR Clinical Cases and 100 Cases in Psychiatry, incorporate elements of the biopsychosocial model and functional assessment in their discussions. However, the benchmark tasks primarily emphasize diagnostic accuracy, treatment decision-making, and knowledge application. Notably, we do not systematically assess broader integrative or systemic dimensions such as narrative biopsychosocial formulation (holistic case conceptualization integrating biological, psychological, and social factors), detailed functional assessment, or systems-level collaboration (e.g., multidisciplinary team planning), nor do we assess interactive communication or conversational coherence.

#### Clinical task design and implementation

The overall objective of capturing the multifaceted reasoning processes essential to psychiatric practice served as a guide for the creation and execution of the PsychiatryBench clinical tasks. Each task type was designed to assess a distinct aspect of clinical competency, including sequential reasoning, longitudinal management, treatment decision-making, and diagnostic formulation. Real clinical cases were converted into structured question-answer pairs that emulate real-world diagnostic and therapeutic reasoning, using only reliable, expert-validated psychiatric sources. The inclusion of medical history and mental state examination (MSE) elements varies across cases, reflecting the structure of the original source texts rather than deliberate design choices. This variability was retained to preserve the authenticity and instructional style of the reference materials. This section outlines the conceptual rationale, data generation procedures, and task-specific frameworks used to operationalize these competencies, thereby providing a foundation for the systematic, clinically grounded evaluation of LLMs in psychiatry. For additional transparency, representative sample items from multiple PsychiatryBench task types are provided in the Supplementary Information (see section “Samples from dataset and responses”).

*Diagnosis:* This subset contains 467 expert-annotated question-answer pairs centered on diagnostic reasoning. The task focuses on determining psychiatric diagnoses based on rich clinical vignettes that simulate real-world case presentations. Each item includes a clinical vignette and a diagnostic query, requiring inference of the most appropriate psychiatric diagnosis. These scenarios often incorporate elements such as the patient’s psychiatric and medical history, presenting symptoms, findings from the MSE, and physical examination. The cases, representing 123 unique diagnostic questions and 295 distinct clinical histories, require models to identify either a likely diagnosis or generate a differential diagnosis, drawing on a nuanced understanding of symptom patterns and temporal progression. Examples are drawn from six authoritative sources, including 100 Cases in Psychiatry and Case Files Psychiatry. This task challenges models to interpret and synthesize complex narratives and apply diagnostic reasoning aligned with formal criteria from standard classifications like the DSM-5-TR, making it critical for evaluating systems intended for clinical decision support and medical education.

*Treatment:* This subset comprises 258 expert-annotated clinical question-answer pairs focused on treatment decision-making. The task centers on identifying appropriate treatment strategies based on clinical scenarios that include a detailed case history, a MSE, physical examination, and a treatment-related question. The expert-curated answers reflect real-world psychiatric care and cover a wide range of disorders and treatment modalities, including pharmacological interventions (e.g., prescribing antidepressants, antipsychotics, mood stabilizers), psychotherapeutic approaches (e.g., cognitive behavioral therapy, psychoeducation, motivational interviewing), and acute care level decisions such as hospitalization when risk severity or treatment resistance requires intensive monitoring and intervention. Sourced from texts like 100 Cases in Psychiatry and Geriatric Psychiatry, this task tests a system’s ability to apply evidence-based psychiatry and personalize care according to clinical guidelines.

*Treatment follow-up:* This dataset comprises 27 clinical question-answer pairs focused on the longitudinal management of psychiatric patients after an initial treatment decision has been made. Each scenario includes a psychiatric case history, a primary follow-up question, and often one or more follow-up reports (e.g., new symptoms, lab results), with a subset of 6 entries extending to a second-tier follow-up. These items evaluate a model’s capacity to support ongoing clinical care by interpreting medication effects, identifying emerging side effects, assessing adherence, or recommending adjustments to the treatment plan. The task simulates ongoing psychiatric care, testing a model’s ability to adapt recommendations over time and handle the incomplete, evolving information that is critical for chronic care planning.

*Classification:* The classification component was developed to evaluate the ability of LLMs to categorize psychiatric disorders based on clinical descriptions. It includes 418 psychiatric case descriptions, each annotated with both a broad diagnostic category and a specific disorder label. Clinical case samples were manually extracted from authoritative casebooks and annotated with preliminary labels. To standardize terminology, two reference lists were created from the DSM-5-TR: one for high-level diagnostic categories (e.g., “mood disorders”) and another for specific disorders (e.g., “bipolar I disorder”); Supplementary Information (see section “Classification Mapping”) A for the full lists. A mapping process was then applied to align each sample with both the validated category-level and disorder-level labels, enabling multi-label classification. Both lists were checked for clinical accuracy by a licensed psychiatrist.

*Management plan:* This dataset consists of 337 case-based question-answer pairs that test a model’s ability to generate a structured and contextually appropriate management plan. Each scenario provides a detailed clinical history, often supplemented with MSE and physical examination findings, followed by a question like “How would you manage this case?” The answers outline holistic, step-by-step care strategies that integrate diagnostic clarification, risk assessment, therapeutic planning (pharmacological and psychological), psycho-education, social interventions, and follow-up. The task emphasizes comprehensive care planning and management decision-making beyond single diagnoses or treatment choices, reflecting the complex care coordination required in real-world psychiatric settings.

*Clinical approach:* The development of this subset began with extracting patient case scenarios from psychiatric reference books. Since these source vignettes were not accompanied by ready-made questions, it was necessary to transform them into evaluable QA pairs. For this purpose, these cases were used to generate open-ended clinical reasoning questions using the Gemini 2.5 Pro model, which was instructed to formulate questions requiring multi-step interpretive reasoning. Subsequently, each generated question underwent manual verification to ensure its clinical relevance. This subset contains 56 expertly crafted scenarios designed to evaluate a model’s ability to emulate the diagnostic thought process of a psychiatrist. Unlike tasks that ask for a final diagnosis, this component focuses on process-oriented reasoning, assessing how clinicians should gather information, rule out critical disorders, prioritize differential diagnoses, and choose initial interventions. The questions emphasize the rationale behind clinical decisions, such as identifying red flags or determining the most appropriate investigative step. This task is essential for assessing how well LLMs can replicate the nuanced cognitive workflow of trained psychiatrists. Unlike tasks that ask for final diagnoses or treatments, this task emphasizes process-oriented clinical reasoning how clinicians gather information, rule out critical disorders, prioritize differential diagnoses, and choose appropriate initial interventions.

*Mental QA:* This dataset includes 326 expert-annotated question-answer pairs that test foundational psychiatric knowledge through the definition and clarification of core concepts, syndromes, and clinical terms. Questions, sourced primarily from the Case Files and Case Vignettes series, ask models to define terms like “bizarre delusions,” explain concepts such as “thought withdrawal,” or describe pharmacological classes. This task targets the factual psychiatric knowledge and terminology aligned with the DSM-5-TR and formal training curricula, enhancing conceptual grounding and promoting explainability in systems intended for clinical education or knowledge base construction.

*Sequential question answering:* This task simulates dynamic, case-based diagnostic reasoning through a dataset of 32 clinical vignettes, each accompanied by a structured sequence of interrelated questions. These questions were designed to reflect the natural progression of a psychiatric evaluation, encompassing five core domains of clinical reasoning: (1) differential diagnosis, (2) supporting evidence, (3) etiological factors, (4) treatment options, and (5) prognosis. To extend the evaluative scope of the dataset, we introduced an additional sixth question classification based on DSM-5-TR, which is not conventionally included in standard psychiatric textbooks. This item was purposefully developed to facilitate diagnostic classification, enabling each vignette to be mapped to a unique label representing the preferred diagnosis. This multi-turn structure provides a rigorous framework for assessing a model’s capacity for longitudinal reasoning, contextual retention, and adaptive clinical judgment across sequential interactions.

*Question formats and exam simulations:* This task group encompasses a variety of structured assessment formats commonly used in psychiatric board examinations and educational settings to assess knowledge recall, clinical recognition, and treatment matching. The development process for all items involved manually curating questions from reputable psychiatry resources and implementing a dedicated preprocessing step to remove all accompanying explanatory text, ensuring an unbiased evaluation format.

*Standard multiple-choice questions (MCQ):* This component consists of 1353 standard multiple-choice questions, each with 4–5 answer options. These items reflect the structured testing style common in psychiatric board examinations and are designed to function as clinical knowledge checks grounded in patient histories and symptom-based reasoning. For instance, a question might describe a patient with specific movements and a family history of psychiatric illness, requiring the identification of a gene location associated with a particular disease. As noted in the introductory description for this section, a dedicated preprocessing step was implemented. Many of the original source items were accompanied by detailed explanations or rationales, which, while pedagogically useful, could introduce bias in LLM evaluation. To prevent this, all explanatory text was removed, ensuring only the question stem and options were retained for an unbiased evaluation. Furthermore, all answer formats were standardized from various original formats (e.g., Roman numerals) into a consistent A/B/C/D labeling scheme, and ambiguous or duplicate questions were filtered to maintain quality.

*Extended matching items (EMI):* This task is designed to assess deeper clinical reasoning by requiring models to match multiple clinical vignettes to a single, shared list of 8-15 answer options (the “theme”). This format tests pattern recognition and fine-grained differentiation across closely related disorders or treatments. The dataset contains 277 whole EMI clusters. To support more granular evaluation and fine-tuning, the full EMI format was also disaggregated into 1037 standalone “EMI separated” question items. Each separated item retains its original header and option list for context but functions as an independent unit, simplifying scoring and improving input granularity during training.

*Exam simulations:* This component includes curated exam-style datasets designed to simulate standardized testing conditions and replicate the structure of real psychiatric board exams. This set contains 600 questions split into two parts for evaluation: 300 EMI questions and 300 MCQs. These items are designed to assess factual recall and decision-making across a complete test, allowing for the evaluation of a model’s performance in a sustained, exam-like scenario.

### Experimental design and models

This subsection outlines the experimental framework used to evaluate the performance of LLMs across the aforementioned tasks (as defined in Section), which simulate real-world clinical workflows. We describe the models employed, including both general-purpose LLMs and domain-specialized medical models. The evaluation methodology combines key implementation details such as prompting and output parsing, automatic metrics, and LLM-as-a-judge assessments to capture performance on accuracy, reasoning depth, and clinical alignment.

#### Language models under evaluation

In this section, we detail the LLMs evaluated throughout our study, covering both general-purpose and domain-specialized systems. The goal is to assess their capabilities in handling various clinically relevant tasks (see Section for task definitions), such as diagnosis, treatment planning, and diagnostic reasoning. By comparing general LLMs with medical-domain-specific models, we aim to highlight the performance trade-offs and advantages of each category. This dual perspective provides a clearer understanding of how LLMs can be optimized or selected for different healthcare applications.

#### General Models

Table 5 presents a concise overview of the LLMs evaluated in the study. It categorizes each model by name, parameter size, source type (open or closed), and the API service provider used to access them.The models include both proprietary and open-source systems. Proprietary models, such as various versions of Google’s Gemini (Gemini 2.5 Pro Preview, Gemini 2.5 Flash “Thinking,” and Gemini 2 Flash), Anthropic’s Claude series (Claude Sonnet 4, 4.5, and 4.5 Thinking), and OpenAI’s GPT 5 Medium Thinking, are closed-source and there is no official public data on model size. As their architectures and training details are not publicly available, they are accessed through their respective API services. Models with ’Thinking’ in their name (e.g., GPT 5 Medium Thinking) may be referred to with a (T) suffix for brevity.

**Table 5:** Summary of LLMs used in our experiments

Model	Size (Parameters)	Source	API service provider	Release (year and quarter)
Gemini 2.5 Pro Preview 03-25^52^	Proprietary	Closed-source	Google’s Gemini API	2025 Q1
Gemini 2.5 Flash Preview 04-17 Thinking^52^	Proprietary	Closed-source	Google’s Gemini API	2025 Q2
Gemini 2.5 Flash Preview 04-17^52^	Proprietary	Closed-source	Google’s Gemini API	2025 Q2
Gemini 2 Flash^52^	Proprietary	Closed-source	Google’s Gemini API	2025 Q1
Claude Sonnet 4^53^	Proprietary	Closed-source	Anthropic API	2025 Q2
Claude Sonnet 4.5^53^	Proprietary	Closed-source	Anthropic API	2025 Q3
Claude Sonnet 4.5 Thinking^53^	Proprietary	Closed-source	Anthropic API	2025 Q3
GPT 5 Medium Thinking^54^	Proprietary	Closed-source	OpenAI API	2025 Q3
DeepSeek-R1^55^	671B	Open-source	DeepSeek API	2025 Q1
DeepSeek Chat^55^	67B	Open-source	DeepSeek API	2024 Q4
LLaMA 3.3 70B^56^	70B	Open-source	Together API	2024 Q4
QWQ 32^57^	32B	Open-source	Nvidia API	2025 Q1
Qwen 3 32B^57^	32B	Open-source	Deepinfra API	2025 Q2
GPT-OSS^56^	120B	Open-source	Together API	2025 Q3

On the other hand, the study includes open-source models like DeepSeek-R1 (671B), DeepSeek Chat (67B), LLaMA 3.3 70B, QWQ 32 (32B), Qwen 3 32B, and GPT-OSS (120B). These models offer more transparency and are made available through platforms such as the DeepSeek API, Together API, Nvidia API, and Deepinfra API. These models have sizes ranging from 32B-671B parameters. Overall, the table illustrates a diverse selection of LLMs sourced from both major tech companies and open research communities, providing a broad basis for comparative experimentation and analysis.

#### Medical models

To ensure a well-rounded and insightful evaluation of LLMs in the context of psychiatry, we selected a diverse group of medical-focused models that reflect a range of architectures, parameter scales, and accessibility levels, as shown in Table 6. This curation was designed to balance frontier performance with practical considerations such as openness, reproducibility, and ease of deployment.

**Table 6:** Summary of medical models used in our experiments

Model	Size (Parameters)	Source	API service provider	Release (year and quarter)
Llama3-OpenBioLLM-70B^58^	70B	Open-source	Hugging Face	2024 Q2
Palmyra-Med-70B^59^	70B	Open-source	Hugging Face	2025 Q1
Llama-MedX v3.2^60^	70B	Open-source	Hugging Face	2025 Q1
JSL-MedLlama-3-8B-v2.0^61^	8B	Open-source	Hugging Face	2025 Q2
MedGemma^62^	27B	Open-source	Hugging Face	2025 Q2
II-Medical-8B^63^	8B	Open-source	Hugging Face	2025 Q3
Med-PaLM (v2)^64^	Proprietary	Closed-source	Google Cloud Vertex AI	2023 Q1

Our model set includes high-capacity open-source systems like Llama3-OpenBioLLM-70B, Palmyra-Med-70B, Llama-MedX v3.2, and MedGemma, which leverage recent advances in foundational models and are optimized for biomedical and clinical tasks. We also included JSL-MedLlama-3-8B-v2.0, a smaller model aimed at providing strong performance while remaining lightweight and adaptable. These open-source models are readily accessible via platforms such as Hugging Face and are suitable for both academic research and fine-tuned clinical deployments.

In addition, we explicitly report each model’s Release (Year and Quarter) to contextualize performance within the rapidly evolving landscape of medical-oriented LLMs. Because model capabilities improve significantly over short development cycles, situating each system within its temporal release window enables more meaningful comparisons. This temporal framing highlights trends such as the recent surge of high-capacity open-source biomedical models, while also clarifying how older, proprietary systems like Med-PaLM (v2) continue to serve as reference benchmarks. Including release timing therefore supports a more accurate interpretation of model maturity, recency, and relevance for clinical and educational applications.

In contrast, we also evaluated Med-PaLM (v2), a proprietary model developed by Google DeepMind. While not open-source, Med-PaLM (v2) represents a high-performing benchmark for medical reasoning and is accessible through Google Cloud’s Vertex AI platform. Its inclusion provides an important comparison point for understanding the trade-offs between commercial-grade deployment and the flexibility of open systems.

By including both open and closed models of varying sizes, we aim to assess not only raw accuracy on psychiatric tasks but also broader factors such as infrastructure requirements, cost, adaptability, and transparency, key considerations for real-world clinical and educational use. A summary of these models, along with their sources and access points, is provided in Table 6.

#### Implementation details

We designed PsychiatryBench to ensure fair, consistent, and reproducible evaluation across models. This section outlines key implementation choices, including how prompts were standardized and how model outputs were parsed. These steps were essential for reliable cross-model comparison given the varied response behaviors of modern LLMs.

#### Prompting strategy

All models were evaluated using a standardized zero-shot prompting approach. While the same prompt format was applied uniformly across all models to ensure fairness in comparison, prompt design was tailored to each specific task type within PsychiatryBench. For example, diagnostic reasoning tasks employed structured clinical case prompts, whereas factual knowledge tasks, such as Mental QA used direct definition queries. This task-specific prompting allowed us to preserve the integrity and intent of each evaluation domain while maintaining cross-model consistency.

Across the benchmark, every task was framed as a combination of prompt prediction and prompt evaluation, ensuring that models were not only generating responses but also being assessed on their ability to interpret and follow clinical or knowledge-based instructions precisely.

We conducted two dedicated guidance sessions to refine the prompt templates used for each category. These sessions explored alternative phrasing, instruction clarity, and structural variations. After comparative analysis, we selected the prompt configuration that produced the highest performance while maintaining conceptual validity. Detailed descriptions of the guidance sessions, including the candidate prompt variations, are provided in Supplementary Information (see section “Prompt templates”).

For evaluation, certain tasks employed an LLM-as-a-judge framework to assess answer quality, particularly for open-ended reasoning or multi-step justification tasks. This approach ensured scalable and consistent scoring while reducing manual annotation demands. The judging rubric, reliability checks, and model parameters used in this process are detailed in Supplementary Information (see section “Prompt templates”).

##### Parsing model outputs

The process of interpreting and extracting meaningful information from LLM outputs emerged as a critical and non-trivial component of our methodology. While it may appear straightforward for tasks involving simple responses such as multiple-choice questions (MCQs) or numerical ratings, the actual output behavior of large models introduced substantial complexity.

Advanced reasoning-oriented models, such as DeepSeek Chat, Gemini Thinking, and Sonnet 4.5 Thinking, often diverged from strict output formats. Rather than outputting a clean label or value, they frequently embedded the answer within lengthy justifications or explanations. This behavior required parsing not just the final answer, but also understanding the structure of the response and retaining the embedded reasoning, which we refer to as “*printing the reasoning*.”

Consequently, our methodology included customized parsers and heuristics tailored to each model’s output style, allowing us to isolate the relevant information while preserving contextual evidence to support qualitative evaluation.

#### Evaluation framework

To assess the performance and alignment of model outputs, we employ a set of evaluation metrics designed to quantify agreement, consistency, and quality across different prompts and models. These metrics allow us to systematically compare outputs, identify patterns in model behavior, and measure improvements across prompt variations. In particular, we focus on agreement counts between model pairs to capture alignment and shared reasoning under different prompting conditions.

*Accuracy* Accuracy is the proportion of correctly classified samples among the total number of samples. It provides an overall measure of correctness; however, it may be misleading in the presence of class imbalance, as it can be biased toward the majority class.

1 $${\mathrm{Accuracy}}=\frac{{\mathrm{Number}}\,{\mathrm{of}}\,{\mathrm{correct}}\,{\mathrm{predictions}}}{{\mathrm{Total}}\,{\mathrm{number}}\,{\mathrm{of}}\,{\mathrm{predictions}}}$$

##### Extended matching item evaluation

In the EMI set, each question consists of multiple subquestions, all of which must be answered based on shared response options. To ensure accurate data evaluation, the dataset was assessed in two distinct formats: first, as a complete EMI set (full-format evaluation), and second, by treating each subquestion as an independent query with its evaluation (separated format).

In the full EMI set format, scoring follows the Partial Scoring System (PCS), which assigns partial credit based on the proportion of correctly answered subquestions in a set. In contrast, the separated subquestion format evaluates each subquestion independently using a binary scoring system. For both formats, any instance of multiple answers being given for a single question is treated as incorrect and scored as zero. The evaluation is done as follows:

*1. Partial Scoring System (PCS) - Full EMI Set Evaluation:* This evaluation method measures how well the model performs on grouped EMI questions, where each set consists of multiple related subquestions. The scoring system allows for partial credit, ensuring that models are not penalized excessively for a few incorrect subanswers. The score for the *i*-th EMI set is computed as the proportion of correct subquestions:

2 $$EMI\_Scor{e}_{i}=\frac{1}{{S}_{i}}\mathop{\sum }\limits_{j=1}^{{S}_{i}}{c}_{ij}$$

where *S*_*i*_ is the number of subquestions in the *i*-th EMI set, and *c*_*ij*_ ∈ {0, 1} is a binary indicator denoting whether the *j*-th subquestion in the *i*-th set is answered correctly. The overall EMI accuracy is then calculated as the mean score across all EMI sets:

3 $$EMI\,\_Accuracy=\frac{1}{N}\mathop{\sum }\limits_{i=1}^{N}EMI\_\,Scor{e}_{i}$$

where *N* is the total number of EMI sets.

*2. Individual Subquestion Evaluation - Separated EMI:* This approach evaluates each subquestion independently, allowing for standard binary scoring per subquestion. It offers a fine-grained view of model performance without being affected by the structure of the EMI sets. The overall accuracy is then computed as:

4 $${\mathrm{Subquestion}}\_{\mathrm{Accuracy}}=\frac{1}{T}\mathop{\sum }\limits_{k=1}^{T}{a}_{k}$$

where *T*: Total number of individual subquestions across all EMI sets and equals ∑_*i*_*S*_*i*_, and *a*_*k*_ ∈ {0, 1}: Binary indicator of correctness for the *k*-th subquestion when evaluated independently.

*Answer Integrity Rule - Penalty for Multiple Answers:* In both evaluation methods, if the model produces multiple answers for a single question (whether set-based or individual), the answer is considered invalid and marked as incorrect. This ensures clarity, fairness, and aligns with human assessment standards.

5 $${c}_{ij}=0\,{\mathrm{if}}\,{\mathrm{multiple}}\,{\mathrm{answers}}\,{\mathrm{are}}\,{\mathrm{provided}}$$

*Common Correct:* This measures the number of questions answered correctly in both formats:

6 $${\rm{C}}{\rm{o}}{\rm{m}}{\rm{m}}{\rm{o}}{\rm{n}}\,{\rm{C}}{\rm{o}}{\rm{r}}{\rm{r}}{\rm{e}}{\rm{c}}{\rm{t}}=\mathop{\sum }\limits_{i=1}^{N}{\bf{1}}({F}_{i}=1\wedge {S}_{i}=1)$$

where *F*_*i*_ and *S*_*i*_ are indicator variables for *Full format* response and *Separated format* response on question *i*, respectively. *F* = 1 if the model answers question *i* correctly in the Full format and 0 otherwise. *S*_*i*_ = 1 if the model answers question *i* correctly in the Separated format and 0 otherwise. ∧ : The logical AND operator. And, **1**(⋅) is the indicator function that returns 1 if the condition inside is true and 0 otherwise.

*Common Incorrect:* This measures the number of questions answered incorrectly in both formats:

7 $${\rm{C}}{\rm{o}}{\rm{m}}{\rm{m}}{\rm{o}}{\rm{n}}\,{\rm{I}}{\rm{n}}{\rm{c}}{\rm{o}}{\rm{r}}{\rm{r}}{\rm{e}}{\rm{c}}{\rm{t}}=\mathop{\sum }\limits_{i=1}^{N}{\bf{1}}({F}_{i}=0\wedge {S}_{i}=0)$$

*Overall consistency:* This score reflects how often the model yields the same result (correct or incorrect) across both formats:

8 $$Consistency=\frac{1}{N}\mathop{\sum }\limits_{i=1}^{N}{\bf{1}}({F}_{i}={S}_{i})$$

*Format Agreement Rate:* This metric calculates the proportion of questions for which the model was correct in both formats, relative to all cases where the model was correct in at least one:

9 $$Agreement=\frac{C}{C+{O}_{F}+{O}_{S}}$$

where *C* is the number of the correct questions, questions the correct questions in both formats, *O*_*F*_ are the correct questions the correct questhetions only in Full format, and *O*_*S*_ are the correct questions the correct questions only in the separated format.

*Format Divergence Rate:* This captures how often the model’s correctness depends on format. A high divergence suggests inconsistent reasoning between question structures:

10 $$Divergence=\frac{{O}_{F}+{O}_{S}}{N}$$

These consistency metrics are crucial for evaluating the robustness and fairness of models, especially in educational and clinical settings where presentation format should not influence correctness.

##### Classification evaluation

Since the classification task is multi-label in nature, each instance may be associated with multiple correct labels. To accurately assess model performance in this setting, we employed two complementary evaluation metrics: *Subset Accuracy and Weighted-Average F1-Score*. These metrics together offer a balanced view rewarding exact correctness while also accounting for partial matches and class imbalance^41^.

*Subset accuracy (Exact Match Ratio):* Subset accuracy is a stringent metric that only considers a prediction correct if the predicted label set exactly matches the true label set no missing or extra labels are allowed. This makes it particularly valuable when precision in full-label prediction is critical. It answers the question: “How often did the model get everything exactly right?”

11 $$Subset\,Accuracy=\frac{1}{N}\mathop{\sum }\limits_{i=1}^{N}{\bf{I}}({\widehat{Y}}_{i}={Y}_{i})$$

where *N* is the total number of samples, *Y*_*i*_ is the set of true labels for the *i*-th sample, ${\widehat{Y}}_{i}$ is the set of predicted labels, **I**(⋅) is the indicator function, and equal to 1 if ${\widehat{Y}}_{i}={Y}_{i}$, and 0 otherwise.

*Weighted-average F1-score (F1-weighted):* While subset accuracy requires perfect prediction, the F1-weighted score provides a more flexible and balanced view of model performance. It evaluates how well the model balances *precision* (avoiding false positives) and *recall* (avoiding false negatives) for each label independently. Then, it aggregates these per-label F1-scores into a single number using a weighted average, where more common labels contribute more to the final score. First, the F1-score for each label *l* is calculated as the harmonic mean of precision (*P*_*l*_) and recall (*R*_*l*_):

12 $${F}_{1,l}=2\times \frac{{P}_{l}\times {R}_{l}}{{P}_{l}+{R}_{l}}$$

Then, the overall weighted-average F1-score across all labels ${\mathcal{L}}$ is computed as:

13 $${\rm{F}}1\,-\,\mathrm{weighted}=\mathop{\sum }\limits_{l\in {\mathcal{L}}}{w}_{l}\cdot {F}_{1,l}$$

Where ${\mathcal{L}}$ is the set of all labels, and *w*_*l*_ is the proportion of samples in which label *l* appears in the ground truth. This metric ensures that performance on frequent labels contributes more heavily, helping to mitigate distortions caused by class imbalance.

##### LLM as a judge

To quantitatively evaluate the output quality of model-generated answers against ground truth references, we employed an automated approach leveraging an LLM as an expert judge^42^. Specifically, Llama 3 70B was configured for this evaluation task. The core objective was to compute a similarity score, ranging from 0 to 100, representing the degree of substantive congruence between the model-generated response (referred to as the *Candidate Answer*) and the reference ground truth (referred to as the *Reference Answer*). Importantly, the evaluation was designed to focus on the core clinical content, including reasoning, correctness, and completeness of the response while disregarding differences in writing style or phrasing for more details see Supplementary Information (see section “Models used for evaluation judging”).

Recognizing the sensitivity of LLM evaluations to prompt design, we systematically compared two distinct evaluation prompts generated by GPT-4.5 and Gemini 2.0 Pro. The GPT-4.5 prompt employed a rigid point-based scoring rubric with specific weights allocated to different content components (full prompt in Supplementary Information; see section “Prompt Templates”), while the Gemini-generated prompt adopted a logic-driven, few-shot learning approach emphasizing holistic understanding of clinical reasoning and semantic meaning (full prompt in Supplementary Information; see section “Prompt templates”). After empirical testing with LLaMA 3.3 70B as the judge model, we selected the Gemini-generated prompt for our final evaluation pipeline. This decision was driven by its superior consistency and reliability across diverse psychiatric tasks, with the logic-driven, example-based approach eliciting more reliable similarity scores compared to the rigid, point-based system.

To identify the most reliable judge model, we conducted a comparative evaluation using three candidate LLMs: LearnLM (J1), LLaMA 3.3 70B (J2), and GPT-4o mini (J3). As detailed in Supplementary Information (see section “Models used for evaluation judging”), LLaMA 3.3 70B consistently assigned scores with higher discernment and sensitivity across diverse model outputs, demonstrating both consistency in ranking and meaningful differentiation of clinical quality. Consequently, LLaMA 3.3 70B was selected as our primary evaluator and remained constant throughout all experiments to eliminate evaluator-induced bias.

All generated outputs for open-ended tasks were evaluated using this single, standardized framework. Each output-reference pair was scored using the same prompt-generated similarity rubric to ensure methodological consistency across the entire benchmark. Individual similarity scores *S*_*i*_ ∈ [0, 100] obtained for each task instance i are then aggregated to compute the final evaluation score by taking the arithmetic mean across all evaluated instances.

Once individual similarity scores *S*_*i*_ ∈ [0, 100] are obtained for each task instance *i*, we compute the *final evaluation score* by taking the arithmetic mean across all evaluated instances:

14 $$Final\,Score=\frac{1}{N}\mathop{\sum }\limits_{i=1}^{N}{S}_{i}$$

where *N* is the number of datasets used. This final score serves as a robust metric for quantifying the overall content-level alignment between model-generated outputs and expert-authored references across multiple clinical domains.

Taken together, these components establish PsychiatryBench as a rigorously curated, methodologically transparent benchmark for evaluating psychiatric reasoning in LLMs. By integrating expert-derived, clinically grounded QA datasets spanning eleven complementary task types with a diverse suite of general-purpose and medical-domain models, standardized task-specific prompting, and carefully engineered output-parsing procedures, our framework is designed to support fair and reproducible comparison across systems. The combination of accuracy-based metrics, specialized scoring schemes for complex formats such as EMIs, multi-label classification metrics, and an LLM-as-a-judge similarity assessment enables both fine-grained and holistic evaluation of content alignment, reasoning depth, and clinical relevance. This methodological foundation provides a robust basis for the subsequent Results and Discussion, where we analyze performance patterns across tasks and models, highlight strengths and limitations, and outline implications for the safe and effective integration of LLMs into psychiatric education and decision support.

## Supplementary information

Supplementary Information

## Data Availability

Datasets generated during the current study are available from the corresponding author on reasonable request.

## Code availability

The code used for the analysis in this study is available upon reasonable request.
