# Supplementary material for: PsychiatryBench: a multi-task benchmark for LLMs in psychiatry
Source: NPJ Digit Med. 2026 Apr 14;9:320. doi: 10.1038/s41746-026-02582-w (PMC13087022; doi:10.1038/s41746-026-02582-w)
Supplement: Supplementary file 1 — Supplementary Information [file 41746_2026_2582_MOESM1_ESM.pdf]

Supplementary Materials for PsychiatryBench: A Multi-Task Benchmark for LLMs in Psychiatry

1 Extended Evaluation Details

1.1 Extended Matching Item and Separated Extended Matching Item

A methodological point worth noting is how we handled EMI items that appeared in both the full-set format and the separated-subquestion format. Although the original EMI dataset contained 277 extended matching sets, not all of these sets were represented in a fully comparable way across the two evaluation formats. In practice, some EMI sets appeared only in the full-format runs or only in the separated runs, and in a few cases the subquestion structure or answer keys were not perfectly aligned between formats. In addition, response options in the model outputs sometimes differed only in letter case (e.g., ”a” vs. ”A”); to avoid spurious mismatches, all responses and answer keys were case-normalized before scoring. To avoid introducing bias from missing, structurally mismatched, or trivially misaligned items, we therefore restricted our consistency and format-sensitivity analysis to the subset of EMI sets that were present and structurally compatible in both conditions. This yielded a shared subset of $N = 144$ EMI sets with a total of $T = 514$ overlapping subquestions, for which we could compute correctness in the full format (F_i) and the separated format (S_i) using the same answer key and the same Answer Integrity Rule.

Focusing on this overlapping subset has two important implications. First, it strengthens the internal validity of the comparison between formats: every metric Total Correct (Full / Separated), Common Correct, Common Incorrect, Total Incorrect (Full / Separated), Average Partial Credit Scores (PCS) in both formats, Accuracy (Separated), Overall Consistency, Format Agreement Rate, and Format Divergence Rate is computed on exactly the same pool of underlying subquestions (Supplementary Tables 1 and 2). This ensures that any observed differences are attributable to differences in presentation format and model behavior, rather than to differences in item composition across conditions. Second, this restriction necessarily means that our format-comparison results should be interpreted as applying to the shared core of the EMI dataset rather than to all 277 sets. The excluded items are not assumed to be easier or harder, but they fall outside the strict intersection required for a one-to-one comparison. Overall, this design choice reflects a trade-off between breadth and fairness: by sacrificing some coverage of the original EMI pool, we obtain a cleaner and more conservative estimate of how stable each model's performance is across formats.

A second methodological choice concerns the selection of models included in the comparative analysis. We initially evaluated a larger pool of 15 models on the EMI-Full setup and then restricted our detailed consistency and format-sensitivity analysis to the top-performing systems on this task: GPT-5, Sonnet 4.5 (T), Gemini 2.5 Flash (T), Gemini 2.5 Pro, Gemini 2.5 (T), and DeepSeek-R1. This decision reflects both practical and scientific considerations. From a practical standpoint, these are precisely the models that are most likely to be considered for deployment in educational or clinical assessment contexts; understanding their robustness to format changes is therefore the most consequential. From a scientific standpoint, concentrating on the strongest systems allows us to probe whether high aggregate performance on EMI-Full (as quantified by PCS over all 277 sets) necessarily translates into stable behavior under the more granular Separated evaluation. The resulting comparison is thus not intended as an exhaustive benchmark of all available models but as a targeted analysis of the current leading systems under a unified evaluation framework (PCS-based EMI-Full scores and subquestion-level Separated scores on the 144-set overlap.

The numerical results for this overlapping subset, as reported in Supplementary Tables 1 and 2, reveal several notable patterns. On the original 277-set EMI-Full benchmark, average PCS is already high for all selected models, ranging from 85.8% for DeepSeek-R1 up to 89.1% for GPT-5, with intermediate values for Gemini 2.5 Flash (86.1%), Gemini 2.5 Pro (87.9%), and Sonnet 4.5 (T) (88.9%). When we restrict attention to the 144 overlapping sets, the same ordering largely persists: DeepSeek-R1 drops to (72.0%) PCS in the full format on this subset, whereas Gemini 2.5 Flash (T) reaches (75.7%), Sonnet 4.5 (T) (84.7%)–85.8%), Gemini 2.5 Pro (87.5%), and GPT-5 (87.2%). This pattern suggests that the overlapping subset is broadly representative of the difficulty profile of the full EMI pool, rather than being skewed toward unusually easy or hard items: the strongest models remain strong, and the weaker one (DeepSeek-R1) still lags behind in full-format PCS.

Second, direct comparison of the full and separated formats shows that top models are generally robust to the shift from extended matching sets to item-by-item subquestions, but not completely insensitive to it. In the separated condition, subquestion-level accuracy on the overlapping subset ranges from (83.6%) for DeepSeek-R1, through (86.0%) for Gemini 2.5 Pro and (87.8%) for GPT-5, up to (88.1%) for Gemini 2.5 Flash (T), with Sonnet 4.5 (T) in the mid-80s (around 86%). Average PCS in the separated format follows a similar pattern: (84.9%) for DeepSeek-R1,

(86.2%) for Gemini 2.5 Pro, (87.8%) for GPT-5, and (89.0%) for Gemini 2.5 Flash (T), again with Sonnet 4.5 (T) very close to the top tier. For some models (notably DeepSeek-R1 and Gemini 2.5 Flash), separated accuracy and PCS are slightly higher than their full-format counterparts on the same overlapping items, suggesting that decomposing complex EMIs into atomic subquestions can help format-sensitive models avoid cross-item interference and better exploit local item cues. For others (Gemini 2.5 Pro, GPT-5, and Sonnet 4.5 (T)), separated performance is extremely close to full-format performance on the overlap, with only modest gains or small trade-offs, consistent with a more stable internal representation of the underlying medical knowledge.

Third, the consistency-oriented metrics make it possible to distinguish genuinely format-stable models from those that merely achieve similar aggregate scores by different patterns of errors. Overall Consistency defined as the proportion of overlapping subquestions that are either correct in both formats or incorrect in both, varies substantially across models. DeepSeek-R1 shows the lowest consistency at (78.3%), whereas Gemini 2.5 Flash (T) achieves (86.7%), Sonnet 4.5 (T) around (80%–92%), Gemini 2.5 Pro (95.7%), and GPT-5 (93.8%). The corresponding Format Divergence Rates span almost an order of magnitude: 0.217 for DeepSeek-R1, 0.133 for Gemini 2.5 Flash (T), around 0.20 for Sonnet 4.5 (T), 0.062 for GPT-5, and only 0.043 for Gemini 2.5 Pro. In other words, Gemini 2.5 Pro and GPT-5 change their correctness status between formats on only about 4–6% of overlapping subquestions, while DeepSeek-R1 does so on more than 20%. Format Agreement measured at the level of the chosen option letter, irrespective of correctness is high across the board (from (85.4%) for Gemini 2.5 Flash (T) up to (95.2%) for Gemini 2.5 Pro), but the gap between agreement and correctness-based consistency reveals how often models "agree with themselves" on a wrong answer versus maintaining both correctness and choice across formats.

Taken together, these findings suggest that strong performance on EMI-Full does not guarantee complete robustness to changes in question format. However, the leading models, especially Gemini 2.5 Pro and GPT-5, display a relatively high degree of format invariance, combining high full-format PCS (87.5%–89.1%) with strong separated accuracies (86.0%–87.8%) and very low divergence (0.043–0.062). From an assessment perspective, this is encouraging: it implies that for top-tier models, extended matching sets and separated-item formats may be used somewhat interchangeably without dramatically altering performance profiles, provided that scoring rules and answer integrity constraints are held fixed. At the same time, the non-trivial divergence observed for models such as DeepSeek-R1 (21.7% divergence) and, to a lesser extent, Gemini 2.5 Flash (T) and Sonnet 4.5 (T), highlights the importance of specifying and standardising evaluation formats when comparing models or using them in educational settings.

Finally, these analyses underscore the value of designing evaluation protocols that explicitly test format sensitivity, rather than relying solely on aggregate scores in a single presentation style. Future work could extend the present approach in several directions: by incorporating a broader range of models (including weaker baselines and smaller open-source systems), by exploring additional EMI transformations (e.g., varying distractor sets, randomising option labels, or altering clinical context), and by examining how format sensitivity interacts with domain shifts and language variation. Such extensions would provide a more complete picture of how large language models internalise and apply domain knowledge and of the conditions under which their apparent competence on structured medical examinations remains stable or begins to fracture under seemingly minor changes in task framing.

Supplementary Table 1. Comparison of EMI full-set vs. separated formats for top models (144 EMI sets, 452 subquestions).

Models / Data	Total Correct (Full)	Total Correct (Separated)	Correct in Both	Incorrect in Both	Total Incorrect (Full)	Total Incorrect (Separated)
DeepSeek-R1	328	378	304	50	124	74
Gemini 2.5 Pro	452	444	437	57	64	72
Gemini 2.5 Flash (T)	362	398	350	42	90	54
Sonnet 4.5 (T)	451	458	433	53	78	71
GPT-5	395	397	382	42	57	55

1.2 Models Used for Evaluation Judging

In our evaluation, the objective was to determine which prompt formulation leads to the highest level of agreement among different language models. The underlying assumption is that a more general and neutral prompt would guide models to produce more aligned outputs, reflecting an interpretation of the input and task requirements. To quantify this, we examined the number of instances where two models produced the same output (i.e., agreements).

Supplementary Table 2. Evaluation of EMI performance metrics for full-set vs. separated formats across top-performing models on a shared subset of 144 EMI sets.

Models / Methods	Avg. PCS Full (277)	Avg. PCS Full (144)	Accuracy (Separated)	Avg. PCS Separated	Overall Consistency	Format Agreement	Format Divergence
DeepSeek-R1	85.8%	72.0%	83.6%	84.9%	78.3%	93.2%	0.217
Gemini 2.5 Pro	87.9%	87.5%	86.0%	86.2%	95.7%	95.2%	0.043
Gemini 2.5 Flash (T)	86.1%	75.7%	88.1%	89.0%	86.7%	85.4%	0.133
Sonnet 4.5 (T)	88.9%	85.8%	86.6%	86.5%	91.9%	90.4%	0.081
GPT-5	89.1%	87.2%	87.8%	87.8%	93.8%	75.6%	0.062

The higher the agreement count between any two models, the more likely it is that the prompt encouraged uniform behavior and interpretation across model architectures. Supplementary Table 3 summarizes the results on three prompts for Supplementary Prompt 1(GPT-4.5-tuned),and Supplementary Prompt 2(basic), and Supplementary Prompt 3(FS), and across four model pairings: LLaMA & GPT, GPT & DeepSeek, DeepSeek & LearnLM, LLaMA & DeepSeek, GPT & LearnLM, and LLaMA & LearnLM.

Supplementary Prompt 1. Prompt generated by GPT-4.5 for point-based similarity scoring of clinical reasoning responses.

```
I will provide two texts: Text A (LLM's response) and Text B (True answer). Evaluate their similarity
based strictly on the presence and accuracy of key diagnoses, differential diagnoses, and clinical
reasoning. Follow these guidelines:
  • Assign a similarity score between 0 and 100:
     - 0: Text A does not contain the correct diagnoses or significantly misrepresents key clinical
     reasoning compared to Text B.
     - 50: Text A identifies some key diagnoses but either misses important differentials or
     partially
     misrepresents clinical
     reasoning compared to Text B.
     - 100 means Text A correctly captures all essential diagnoses,
     differential diagnoses,and clinical reasoning as stated in Text B.
  • Use the following criteria to guide your scoring:
     - Presence of the correct main diagnosis (40 points).
     - Inclusion and accuracy of differential diagnoses (30 points).
     - Accuracy and completeness of clinical reasoning and justification (30 points).
  • Provide ONLY the numeric similarity score (0-100) without any additional explanation or
    justification.
```

Supplementary Prompt 3 (few-shot setting), which includes examples to guide the model while remaining broad in tone, resulted in the highest levels of agreement among model pairs, particularly between DeepSeek and LearnLM (62 outputs), LLaMA and DeepSeek (57 of 66), and LLaMA and LearnLM (59). These results suggest that the few-shot formulation effectively encouraged models to interpret and respond to tasks similarly, promoting cross-model consistency. Supports the idea that prompting strategies incorporating minimal bias and universal structure are better suited for comparative benchmarking involving multiple LLMs.

In contrast, Supplementary Prompt 2 (basic), (structured scoring setting), which was designed based on GPT-4.5 style prompting, produced the lowest agreement scores across nearly all pairings. This discrepancy indicates that prompts optimized for a specific model's reasoning and alignment tendencies may compromise their generalizability when used with other models. For example, while such tailored prompts may boost in-model performance, they introduce implicit cues or formatting expectations that models may interpret differently or even ignore.

In addition to evaluating model agreement under different prompts, we also assessed the reliability and quality of

Supplementary Prompt 2. Basic Evaluation Prompt

```
You are an evaluator. Your task is to compare a Candidate Answer with a Reference Answer and assess
how well the Candidate Answer captures the core diagnostic meaning of the Reference Answer.

Focus on whether the main diagnosis and important differential diagnoses are reasonably represented.
Minor differences in wording, ordering, or level of detail should not affect the score.

Provide a single similarity score between 0 and 100, where higher scores indicate greater overlap in
diagnostic meaning.

Do not provide explanations or justifications. Output only the numeric score.

Reference Answer: {Answer}

Candidate Response: {Response}
```

Supplementary Table 3. Model agreement counts across three prompt types: Supplementary Table 3 summarizes the results across three evaluation prompts: Supplementary Prompt 1 (GPT-4.5-tuned structured scoring), Supplementary Prompt 3 (few-shot, model-agnostic evaluation), and Supplementary Prompt 4 (basic evaluation prompt).. Each value indicates the number of agreeing samples (e.g., "0 of 66"). Higher values suggest greater alignment in model outputs. LLaMA = LLaMA 3.3 70B, DeepSeek = DeepSeek-R1.

Prompt \Models	LLaMA & GPT-4	GPT & DeepSeek	DeepSeek & LearnLM	LLaMA & DeepSeek	GPT & LearnLM	LLaMA & LearnLM
P1	0	0	56	50	0	56
P2	0	0	62	57	0	59
P3	4	3	41	13	9	12

LLM-as-a-judge frameworks using three different evaluators: LearnLM (J1), LLaMA 3.3 70B (J2), and GPT-4o mini (J3). Each judge was tasked with scoring the output of five candidate models: DeepSeek-R1, DeepSeek Chat, Gemini 2 Pro, Gemini 2 Thinking, and QWQ-32 across a set of medical diagnosis tasks derived from expert-authored case studies.

The evaluation results, shown in Supplementary Table 4, reveal that LLaMA 3.3 70B (J2) consistently assigned higher and more discriminating scores compared to the other two judges. While GPT-4o mini produced lower and somewhat conservative scores across all models, and LearnLM showed moderate variance, LLaMA 3.3 70B demonstrated both consistency and sensitivity in differentiating model quality. For instance, it rated DeepSeek-R1 at (73.99) and Gemini 2 Pro at (77.78), capturing meaningful performance differences that aligned with expert review.

These findings suggest that LLaMA 3.3 70B serves as a highly effective automatic evaluator for medical QA tasks. Its strong performance may be attributed to its refined instruction-following abilities and robust handling of domain-specific content. Therefore, LLaMA 3.3 70B was selected as the preferred judge in our broader evaluation framework, as it most accurately reflected human-like judgment in interpreting and scoring clinical reasoning tasks.

1.3 Samples from Dataset and Responses

To offer a transparent view of the evaluation process and the nature of the tasks assessed in this study, we present selected sample cases from four representative task categories: Diagnosis, Treatment, Management Plan, and Sequential Question Answering. These tasks reflect the diversity and complexity of clinical reasoning that LLMs must navigate when applied to mental health contexts.

Supplementary Table 4. Comparison of model scores as evaluated by three different LLM judges (J1: LearnLM, J2: LLaMA 3.3 70B, J3: GPT-4o mini). LLaMA 3.3 70B consistently assigned scores with higher discernment across diverse models, supporting its selection as the preferred evaluator. The underlined bold values are the best score for the specific column.

Judge / Models	DeepSeek-R1	DeepSeek-Chat	Gemini 2 pro	Gemini 2 thinking	QWQ 32
J1(LearnLM)	60.97	70.70	72.32	72.04	72.68
J2(Llama 3.3 70B)	**73.99**	**77.42**	**77.78**	**77.15**	**76.32**
J3(GPT 4o mini)	52.83	61.38	60.73	58.72	56.41

Each sample includes the following components:

- **Book Name:** The medical textbook from which the case was selected.
- **Case Number and Title:** The case ID and title as listed in the original psychiatric textbook. For consistency, shorter and diagnostically focused cases were selected.
- **Case History:** A brief clinical vignette describing the patient's background, symptoms, and context.
- **Mental State Examination (MSE):** A structured assessment of the patient's current psychological functioning, including appearance, behavior, speech, mood, thought processes, perception, cognition, and insight. This component provides essential clinical information for differential diagnosis and management planning.
- **Physical Examination:** A brief summary of relevant physical or neurological findings, included when somatic or organic causes may contribute to psychiatric symptoms. This step helps rule out medical conditions that can mimic or exacerbate mental disorders.
- **Question:** A task-specific query posed to the model, such as identifying the most likely diagnosis or suggesting a suitable management step.
- **Textbook Answer:** The gold-standard or human-annotated answer sourced from the reference material.
- **LLM Response:** The generated output from the best-performing model (based on average score across the evaluation framework).
- **LLM-as-Judge Score:** An automatic evaluation score produced by prompting a strong LLM to assess the response along clinical relevance, correctness, and reasoning.

These examples aim to demonstrate not only the performance of models in isolated tasks but also their behavior in sequential, multi-turn scenarios where context retention and logical flow are critical. All sample cases were sourced from English-language psychiatric textbooks and carefully selected based on clarity, brevity, and relevance to core clinical tasks.

A detailed breakdown of selected examples for each task type is provided below.

Supplementary Prompt 3. Prompt generated by Gemini for Llama 3 70B evaluation.

```
SYSTEM: You are a medical diagnosis evaluator. Your task is to compare two medical text summaries and assess
the degree to which the first text (the "Candidate Answer") captures the essential diagnostic information
contained in the second text (the "Reference Answer"). Focus on the core medical meaning and the completeness
of the diagnoses, not on the writing style or specific wording.
USER:
INSTRUCTIONS:
1. Read the "Reference Answer" carefully. Identify the key diagnoses (both primary and differential) and any
crucial supporting information (e.g., symptoms, test results, ruled-out disorders).
2. Read the "Candidate Answer". The "Candidate Answer" will be a concise list of diagnoses and key findings.
3. Compare the "Candidate Answer" to the "Reference Answer" based on the following criteria:
   • Diagnostic Accuracy: Does the "Candidate Answer" correctly identify the most likely diagnosis
   (if one is clearly indicated in the "Reference Answer")?
   • Diagnostic Completeness: Does the "Candidate Answer" include all of the major differential diagnoses
   mentioned in the "Reference Answer"? It is acceptable if the "Candidate Answer" uses slightly different
   terminology as long as the underlying medical concept is the same.
   • Supporting Information: Does the "Candidate Answer" include the key symptoms, test results,
   or other evidence that support the diagnoses? It doesn't need to include every detail, but it should capture
   the most important ones.It's ok if this supporting information is implicitly present in the reference answer
   • Exclusion of Irrelevant Information:
   Do not penalize the "Candidate Answer" for including additional
   information that is not present in the "Reference Answer", as long as that information is medically accurate
   and relevant to the case.
   • groundance does not affect the score:Do not penalize a response for being redundant
4. Provide a single numerical similarity score between 0 and 100, where:
   • 0: The "Candidate Answer" has no meaningful overlap with the "Reference Answer" in terms of diagnosis or
   supporting information. It's completely wrong.
   • 50: The "Candidate Answer" identifies some relevant diagnoses or supporting information, but it misses
   major elements or includes significant inaccuracies.
   • 75: The "Candidate Answer" correctly identifies the primary diagnosis
   (if applicable) and most of the important differential diagnoses and supporting information. It may miss
   some minor details or include some slight inaccuracies.
   • 90: The "Candidate Answer" is very close to the "Reference Answer" in terms of core diagnostic meaning and
   completeness. It may have minor omissions or differences in emphasis, but it captures almost all of
   the essential information.
   • 100: The "Candidate Answer" is a perfect match in terms of core diagnostic meaning. It includes all of
   the key diagnoses and supporting information from the "Reference Answer",even if the wording
   or organization is different.
5. Do not justify or explain the answer. Give a score between 0 and 100, no other text.
Here is an example for better understanding:
Reference Answer:
Differential diagnosis: 1. Delirium 2. Substance intoxication 3.Substance withdrawal
delirium 4. Mental retardation
Candidate Answer:
- Primary Diagnosis: Alzheimer's_Disease
- Key Findings: Progressive memory loss, disorientation, difficulty with ADLs.
- Differential Diagnoses: Vascular_Dementia,
Lewy_Body_Dementia, Frontotemporal_Dementia
ASSISTANT: 75
Here is another example:
Reference Answer:
Alcohol Use Disorder (Severe)
Candidate Answer:
- Primary Diagnosis: Alcohol_Use_Disorder (Mild)
- Key Findings: History of heavy alcohol use, withdrawal_symptoms.
ASSISTANT: 50
Now, evaluate the following:
Reference Answer : {Answer}
Candidate Response: {Response}
```

1.3.1 Diagnosis Case

Book Name: 100 Cases in psychiatry
Case Number and Title: Case 3 – *Generalized Anxiety*
Case History: A 40-year-old school teacher attends his general practitioner's surgery with his wife, complaining of feeling constantly fearful. These feelings have been present on most days over the past 3 years and are not limited to specific situations or discrete periods. He also experiences poor concentration, irritability, tremors, palpitations, dizziness and dry mouth. He has continued to work, but his symptoms are causing stress at work and at home. He denies any problems with his mood and reports that his energy levels are fine. He admits that he is experiencing problems with his sleep. He finds it difficult to fall asleep and states that he does not feel refreshed on waking up. He has been married for 15 years and lives with his wife and two sons aged 8 and 10. His parents live locally and he has no siblings. His father has been diagnosed with Alzheimer's dementia. He remembers his mother being anxious for much of his childhood. He has no previous medical or psychiatric history and is not taking any medication. He smokes 20 cigarettes per day and drinks alcohol socially. He has never used any illicit drugs. He tends to hide his symptoms and said that he was seeing his GP because his wife wanted him to seek help.
Question: What is the differential diagnosis?

Textbook Answer

- Depression: Anxiety symptoms are common in depression and co-morbid depression is often seen with GAD. The type of symptom that appears first and is more severe is conventionally considered to be primary.
- Panic disorder: There is a discrete episode of intense fear with sudden onset and a subjective need to escape.
- Other anxiety disorders: They have the same core symptoms as in GAD, but the symptoms occur in specific situations, as in phobic anxiety disorder, OCD, or PTSD.
- Substance misuse: Symptoms of alcohol or drug withdrawal may mimic those of anxiety.
- Physical illness: A host of medical conditions can mimic GAD—endocrine disorders such as hyperthyroidism or phaeochromocytoma; neurological disorders such as migraine; deficiency states such as anaemia or vitamin B12 deficiency; cardiac conditions such as arrythmias and mitral valve prolapse, and metabolic conditions such as hypoglycaemia and porphyria.

Sonnet 4.5 Thinking Response

1. Generalized Anxiety Disorder (GAD)
Most likely Supporting features:

- Excessive, persistent worry on most days for 3 years (>6 months required)
- Free-floating anxiety (not situation-specific or episodic)
- Autonomic symptoms: palpitations, tremors, dizziness, dry mouth
- Psychological symptoms: poor concentration, irritability, sleep disturbance
- Significant functional impairment (work and home stress)
- Family history of anxiety in mother

2. Panic Disorder

- Symptoms are constant rather than in discrete panic attacks
- Patient specifically states symptoms are "not limited to discrete periods"
- However, panic disorder can have anticipatory anxiety between attacks

3. Depression (with anxious distress)

- Sleep disturbance (initial insomnia, unrefreshing sleep)
- Poor concentration, irritability
- Patients may not recognize depressive symptoms
- Requires formal screening with PHQ-9 or similar tool

4. Tourette Syndrome or Chronic Tic Disorder

- Facial tic present
- However, typically more persistent rather than episodic

5. Mixed Anxiety-Depressive Disorder)

- Combination of both symptom clusters
- Neither clearly predominant

6.Substance-Induced Anxiety

- Nicotine (20 cigarettes/day) - withdrawal between cigarettes
- Caffeine excess - needs assessment
- Alcohol - pattern unclear; chronic use or withdrawal
- Clarify actual alcohol consumption ("socially" is vague)

Judge Score: 90%

1.3.2 Management Plan

Book Name: 100 Cases in Psychiatry
Case Number and Title: Case 2 – *Untreated dental abscess*
Case History: A 34-year-old woman attends the emergency department of a hospital with a dental abscess. She leaves while waiting for a doctor to come and see her, but returns the same evening. When the doctor arrives she explains that she has a terror of dentists and has not seen one since she was 8 years old. She has several memories of pain while being given fillings. She explains that she was allowed to eat unlimited sweets as a child and that brushing her teeth was not part of a routine established by parents. She started brushing her teeth when she was 14 and became self-conscious of her appearance. She remembers needing to go to the dentist when she was 16 because of a painful tooth. She became very worried for several days, being unable to sleep well and having episodes when she became frightened and breathless. On that occasion she repeatedly refused to see the dentist and was given antibiotics by her GP which settled the infection. On this occasion she has made several appointments to go to the dentist but has either cancelled them or not gone to the appointment. She realizes that she needs treatment and she is clearly in pain but cannot overcome her fear.
Question: What can you do to help?
Mental State Examination: When the doctor arrives she is clearly on edge, and is sweating and shaking. Her pulse when measured is 98 beats/min and her blood pressure is 130/70 mmHg. She is vigilant to sounds and activity around her in the department. There are no thoughts of self-harm and she is able to enjoy herself when at home or with friends and she is not in pain. There is no evidence or history of thought passivity or psychotic phenomena.
Physical Examination: Not Reported

Textbook Answer

This woman has a fear of dentists. This is more than a typical and appropriate anxiety experienced by many people, since it leads to an untreated and potentially serious and painful condition, an abscess.Phobias often have some element of understandable fear such as thunderstorms, dogs, flying, heights, needles and dentists. Many of these can be risky in some situations, although for the most part these experiences in our society are painless and harmless. The fear in phobia is far in excess of that 'usually' experienced. Some phobias are instinctive and are programmed through natural selection. These would include fears of spiders and snakes. Some are associative such as blood (for example, associated with images of harm or injury). Some have none of these factors (for example, buttons, cardboard, glitter, wooden spoons) and may be related to negative early life experiences, for example, being beaten as a child by a wooden spoon. The best treatment for a phobia is desensitization or cognitive behaviour therapy (CBT). The latter will usually include some elements of desensitization alongside psychoeducational strategies. Medication (such as a benzodiazepine) is not usually used in phobias unless it is part of a short-term strategy to enable CBT to start. Desensitization involves exposure to a hierarchy of feared situations drawn up in conjunction with the phobic person. The list is scored for fear, and exposure with support (and sometimes rewards) is systematically worked through. For example, this woman may look at pictures of dentists, videos of a normal dental health check and may visit the dental surgery without any treatment. She may take home dental masks and mouthwash. She may watch someone else having a check and may agree to sit in the dentist's chair and have her mouth examined with no treatment. Imaginary desensitization involves using imagined scenarios in the hierarchy. Relaxation, hypnotherapy and autohypnosis may all give feelings of control to the sufferer and reduce anxiety. Clearly none of this can happen while she has an abscess and this needs to be treated in the first instance. An X-ray may be part of a desensitization list with treatment being performed under general anaesthetic or with sedation. Use of sedation at this point would be to treat the abscess not the phobia and CBT would follow successful treatment of the abscess. In this situation, most areas have specialist dentists (community dental officers) who are used to dealing with phobias and it will be worth arranging an appointment. A psychologist or community mental health nurse will be able to carry out the CBT.

Sonnet 4.5 Thinking Response

The primary diagnosis here is a specific phobia of dental treatment, which has developed following traumatic childhood experiences and has been reinforced through avoidance behavior over many years. The immediate goal is to address both the acute dental abscess and the underlying phobia to prevent future crises. In the immediate term, it is essential to establish a supportive therapeutic relationship by validating her fear and acknowledging that her distress is real and understandable given her history. The acute dental abscess requires medical management with appropriate antibiotics and analgesia to control the infection and pain while a more comprehensive treatment plan is developed. This provides time to implement psychological interventions before definitive dental treatment becomes necessary. The most effective treatment for specific phobias is cognitive behavioral therapy, particularly exposure-based therapy. This would involve gradual, systematic desensitization to dental situations, starting with less threatening exposures such as visiting a dental surgery without treatment, then progressing through a hierarchy of increasingly challenging situations until she can tolerate necessary dental procedures. Cognitive restructuring would help her identify and challenge catastrophic thoughts about dental treatment and replace them with

Sonnet 4.5 Thinking Response Continued

more realistic appraisals. Teaching relaxation techniques and controlled breathing exercises would provide her with tools to manage acute anxiety symptoms when they arise. It is crucial to establish a collaborative approach with dental services, ideally referring her to a dentist who has experience and training in treating highly anxious patients. Many dental practices offer special accommodations such as longer appointments, clear communication about each step of treatment, and the use of sedation options ranging from nitrous oxide to conscious sedation or even general anesthesia for more extensive procedures if psychological approaches alone are insufficient.

Short-term pharmacological support with anxiolytic medication such as a benzodiazepine could be considered for specific dental appointments as an adjunct to psychological treatment, but this should not replace the primary focus on exposure therapy and anxiety management skills. Psychoeducation about the nature of phobias and how avoidance maintains and strengthens fear is important to help her understand why confronting her fear, though difficult, is necessary for long-term resolution.

A referral to clinical psychology or psychotherapy services should be arranged for structured psychological treatment. Regular follow-up appointments should be scheduled to monitor progress, provide ongoing support, and adjust the treatment plan as needed. The ultimate goal is not only to enable her to receive the current necessary dental treatment but to establish a pattern of regular dental care that prevents future crises, breaking the cycle of avoidance and emergency presentations that has characterized her dental care for over two decades.

Judge Score: 90%

1.3.3 Treatment

Book Name: Case file Psychiatry
Case Number and Title: Case 8 – *Social Phobia*
Case History: A 35-year-old man visits a psychiatrist because he is overwhelmingly anxious about a speech he has to make. The man states that he was recently promoted to a position within his company that requires him to speak in front of an audience of approximately 100 people. He says that the first such speech is coming up in 2 weeks and that worrying about it keeps him from sleeping. He knows that his fear is out of proportion, but he is unable to control it. He explains that he has always had trouble with public speaking because he fears that he might do something stupid or otherwise embarrass himself. He has avoided public speaking in the past as much as possible or has spoken in public only before an audience of fewer than 10. Because he knows that he must make the presentation coming up in 2 weeks or he will not be able to keep his new job, he has visited the psychiatrist hoping to find a solution to the problem.
Question:
What are the treatment options open to this patient?
Mental state examination: Not Reported
Patient's Physical Examination: Not Reported

Textbook Answer

Treatment options: Behavioral or cognitive-behavioral therapy is the treatment of choice. A typical treatment regimen involves relaxation training followed by progressive desensitization. Pharmacologic interventions include benzodiazepines or beta-blockers over the short term or an antidepressant such as imipramine.

Sonnet 4.5 Thinking Response

Treatment Recommendation
Based on this patient's clinical presentation, a combined pharmacological and psychotherapeutic approach is most appropriate given both his immediate need and long-term career requirements.
Recommended Treatment Plan:

1. Immediate Management (for 2-week deadline): Beta-blocker (propranolol 10-40 mg) taken 30-60 minutes before the presentation. This patient has performance-type social anxiety with an imminent speaking engagement, and beta-blockers are highly effective for acute performance anxiety by blocking peripheral manifestations (tremor, tachycardia, sweating) without causing sedation that could impair performance.
2. Psychotherapy (first-line, long-term): Cognitive Behavioral Therapy (CBT) with exposure therapy should be initiated immediately. This is the evidence-based first-line treatment for social anxiety disorder. While meaningful improvement may not occur within 2 weeks, starting now will address his long-term need for repeated public speaking in his new role. CBT will help him challenge catastrophic thoughts about embarrassment and gradually build tolerance through exposure.
3. Consider SSRI for ongoing management: If symptoms significantly impair functioning beyond this single event, initiating an SSRI (such as sertraline or paroxetine) would be appropriate for sustained treatment of his underlying social anxiety disorder. However, these medications require 4-6 weeks for therapeutic effect and won't address the immediate deadline.

Rationale: This patient has clear social anxiety disorder (performance type) with longstanding avoidance patterns, intact insight, and both immediate and ongoing occupational demands. The combination addresses his acute crisis while establishing long-term management for his career sustainability.

Judge Score: 95%

1.3.4 Sequential Question Answering

Book Name: Core Clinical Cases in Psychiatry
Case Number and Title: Case 1.4 – *Long history of persecutory ideas*
Case History: A 45-year-old man is referred to the outpatient department by his GP. The man says that over recent months he has become increasingly low in mood. He says that he is under intolerable pressure because of persecution by his neighbours. It transpires that for over 20 years he has had to move house very frequently because someone keeps telling his neighbours that he is a paedophile. He denies this, and has no such convictions. He says that as a result, all of his neighbours are against him and spy on him to try to catch him at it and drive him away. He thought that he had escaped when he moved into his current address 18 months ago, but now his persecutor has caught up with him again. He says that he cannot face moving away again. His GP has confirmed that he has changed address many times over the last decade or so, but has otherwise lead a relatively normal life. He presents as a prickly man, with few friends or social networks.
Question 1: What is the likely differential diagnosis?

Q1: Textbook Answer

Preferred diagnosis: Persistent delusional disorder.
Alternative diagnoses: Personality disorder – probably paranoid. Schizophrenia. Depressive episode. Physical causes of psychosis.

Q1: Gemini 2.5 Pro Response

Preferred diagnosis: Delusional Disorder (Persecutory Type)
Alternative diagnoses: Paranoid Personality Disorder, Schizophrenia, Major Depressive Disorder with psychotic features
The patient's long history of perceived persecution, frequent house moves, and lack of evidence to support his claims suggest a delusional disorder. However, his prickly demeanor and limited social networks could also indicate a paranoid personality disorder. The presence of depressive symptoms and potential for psychotic features also warrant consideration of schizophrenia and major depressive disorder with psychotic features.

Q1: Judge Score: 90%

Question 2: What information in the history supports the diagnosis, and what other information would help to confirm it?

Q2: Textbook Answer

The long-standing nature of his systematized abnormal beliefs suggests a persistent delusional disorder. To confirm the diagnosis, you must establish that the beliefs are delusional and have been present for over 3 months. Such long-standing difficulties may also represent a personality disorder – probably a paranoid personality disorder. If the beliefs are overvalued ideas, personality disorder is likely. However, if the beliefs are delusions, personality disorder cannot be the sole diagnosis, though it is often comorbid with a delusional disorder. Schizophrenia may be excluded by demonstrating the absence of first rank symptoms. Depression may be excluded as a primary diagnosis by demonstrating the absence of a depressive syndrome, or by demonstrating that the psychosis developed before the mood disorder. Physical causes are very unlikely in such a long-standing disorder.

Q2: Gemini 2.5 Pro Response

The patient's consistent and persistent belief that his neighbors are against him and spying on him, despite a lack of evidence, supports the diagnosis of delusional disorder. The fact that he has changed addresses multiple times in an attempt to escape this perceived persecution also suggests a fixed delusional system. Additionally, the patient's denial of being a pedophile and the absence of any convictions suggest that his delusions are not based in reality. To confirm the diagnosis, further information about the patient's mental state, including the presence of any hallucinations, disorganized thinking, or negative symptoms, would be helpful. A thorough psychiatric evaluation, including a mental status examination and a review of his past medical and psychiatric history, would also be necessary. Furthermore, collateral information from the patient's GP, family members, or friends could provide additional insight into the patient's behavior and mental state.

Q2: Judge Score: 90%

Question 3: What might the important aetiological factors be?

Q3: Textbook Answer

Predisposing factors: Persistent delusional disorder commonly occurs in people with an abnormal personality – usually a paranoid personality disorder.
Precipitating factors: It will be difficult to identify precipitating factors, as the symptoms have been present for so long.
Maintaining factors: Maintaining factors related to his personality and lifestyle may be difficult to modify. For example, his persecutory beliefs are likely to increase his isolation from other people, which will in turn fuel his beliefs.

Q3: Gemini 2.5 Pro Response

Predisposing factors: The patient's prickly personality and limited social networks may have contributed to his vulnerability to developing a delusional disorder. A history of trauma or stress, such as being falsely accused of a crime, could also be a predisposing factor.
Precipitating factors: The patient's recent move and perceived renewal of persecution may have triggered the current episode of depression and exacerbation of his delusional symptoms.
Maintaining factors: The patient's social isolation, lack of support network, and repeated reinforcement of his delusional beliefs through his frequent house moves may be maintaining his delusional disorder. Additionally, his avoidance of social interactions and lack of engagement in activities may be perpetuating his symptoms.

Q3: Judge Score: 90%

Question 4: What treatment options are available?

Q4 Textbook Answer

Location: Because of his low mood and persecutory beliefs, you must consider his risk of self-harm and the risk of harm to others. If these are assessed as high, then inpatient treatment may be necessary. He may want to spend a period away from home, to give himself a break from his persecution, though his delusional beliefs may transfer themselves to nurses or other patients in hospital. In practice, outpatient treatment should be possible with close monitoring by a CPN.
Physical: He has a psychosis – therefore, a trial of treatment with an antipsychotic is necessary. If he has a comorbid depressive illness, an antidepressant may be useful in combination.
Psychological: Discouragement of maladaptive coping strategies, such as alcohol misuse or not going out of the house, is an important first step, together with an attempt to establish a trusting relationship. Cognitive therapy may then modify his abnormal beliefs. The aim is probably not to 'cure' him of his beliefs, but to attempt to reduce their intensity and the impact that they have on his life and functioning.
Social: To improve his social networks and reduce his isolation.

Q4: Gemini 2.5 Pro Response

Location-based options: The patient may benefit from a change in environment, such as a move to a new area, to help him escape his perceived persecution. However, given his history of frequent moves, this may not be a viable long-term solution.
Physical treatments: Antipsychotic medication, such as risperidone or olanzapine, may be effective in reducing the patient's delusional symptoms and associated anxiety and depression.
Psychological support: Cognitive-behavioral therapy (CBT) or other forms of talk therapy may help the patient to address his delusional beliefs, develop coping strategies, and improve his social skills and relationships.
Social considerations: The patient may benefit from social support, such as group therapy or support groups, to help him develop a social network and reduce his feelings of isolation. Involvement of his GP and other community mental health services may also be necessary to provide ongoing support and monitoring.
Use of Mental Health Act: If the patient's symptoms worsen or he becomes a risk to himself or others, consideration may be given to using the Mental Health Act to ensure his safety and provide compulsory treatment.

Q4: Judge Score: 90%

Question 5: What is the prognosis in this case?

Q5 Textbook Answer

The prognosis of persistent delusional disorder is poor. The psychosis is likely to remain present indefinitely, despite treatment. Treatment may be successful in reducing his preoccupation with his symptoms. Many patients who present with persistent delusional disorder subsequently develop schizophrenia. Poor prognostic factors include: the length of time the delusions have been present; abnormal previous personality; poor social networks. Good prognostic factors include presence of symptoms of depression, as this can be readily treated.

Q5: Gemini 2.5 Pro Response

Short-term outlook: The patient's prognosis in the short term is guarded, as his delusional symptoms and associated depression may be resistant to treatment. However, with appropriate medication and psychological support, it is possible that his symptoms may improve, and he may be able to develop more adaptive coping strategies.
Long-term outlook: The long-term prognosis for the patient is uncertain, as delusional disorder can be a chronic condition. However, with ongoing treatment and support, it is possible that the patient may be able to manage his symptoms and improve his quality of life. Good prognostic indicators include the patient's relatively normal life prior to the onset of his delusional symptoms and his motivation to seek help. Poor prognostic indicators include the patient's long history of delusional symptoms, his social isolation, and his lack of engagement in activities.

Q5: Judge Score: 90%

Question 6: What is the likely diagnosis?

Q6:Textbook Answer

Persistent delusional disorder

Q6:Sonnet 4.5 Thinking Response

Delusional Disorder (Persecutory Type)

Q6: Judge Score: 100%

Overall Average Judge Score: 90%

Prompt Templates

This details the standardized prompt templates (Supplementary Prompt 1-18) employed across the PsychiatryBench framework to ensure methodological rigor and reproducible evaluation for eleven distinct clinical tasks, ranging from diagnostic reasoning to sequential case analysis. The prompting strategy is bifurcated into prediction prompts, which instruct models to assume a psychiatric persona and generate clinically sound answers based on provided patient history and examination data, and evaluation prompts, which guide the automated "LLM-as-a-Judge" (Llama 3.3 70B) to assign objective similarity scores (0–100) based on strict rubrics of clinical accuracy, completeness, and reasoning alignment relative to expert reference answers.

1.4 Diagnosis Task

To support the diagnostic reasoning process, two structured diagnostic prompts were employed. These prompts were designed to guide the model toward generating clear, consistent, and clinically relevant diagnoses based on the provided case information.

The first prompt focused on diagnostic prediction, explicitly instructing the model to analyze presenting symptoms, relevant clinical history, and contextual factors before proposing a preliminary diagnosis. The second prompt was used for diagnostic evaluation, guiding the model to review and refine the diagnostic conclusion to ensure internal consistency and clinical plausibility.

Together, these prompts enhanced transparency in the diagnostic reasoning process and reduced the risk of unsupported assumptions. The full wording of these diagnostic prompts is provided in Supplementary Prompt 4 - Diagnosis Prediction Prompt and Supplementary Prompt 5 - Diagnosis Evaluation Prompt.

1.5 Treatment Task

Following the diagnostic stage, a treatment-focused prompting strategy was applied. Two structured treatment prompts were utilized: a treatment prediction prompt and a treatment evaluation prompt. The treatment prediction prompt instructed the model to propose an appropriate intervention plan based on the established diagnosis, clinical presentation, and contextual constraints. The treatment evaluation prompt was then used to critically assess the suitability, safety, and clinical relevance of the proposed treatment recommendations.

This two-step approach ensured that treatment suggestions were both theoretically grounded and clinically appropriate. The full wording of the treatment prediction and evaluation prompts is provided in Supplementary Prompt 6 - Treatment Prediction Prompt and Supplementary Prompt 7 - Treatment Evaluation Prompt , respectively.

1.6 Treatment Follow-Up Task

To address follow-up care, two additional prompts were used: a follow-up treatment prediction prompt and a follow-up evaluation prompt. The prediction prompt focused on outlining appropriate follow-up strategies, expected progress, and potential adjustments to the treatment plan. The evaluation prompt assessed the suitability and clinical coherence of the follow-up recommendations. This approach supported continuity of care and ensured alignment with the patient's ongoing clinical needs. These prompts are provided in Supplementary Prompt 8 Treatment Follow-Up Prediction Prompt and Supplementary Prompt 9 Treatment Follow-Up Evaluation Prompt.

1.7 Classification Task

For the classification task, a structured classification prompt was employed to guide the model in assigning cases to predefined diagnostic or clinical categories based on the provided information. The prompt emphasized the use of explicit criteria derived from case features and clinical indicators to ensure consistency and reproducibility. The full wording of the classification prompt is provided in Supplementary Prompt 10 Classification Prompt.

1.8 Management Plan Task

The Management Plan Prediction Prompt (Supplementary Prompt 11) instructs the model to adopt the persona of a psychiatrist and analyze a patient's history, mental state, and physical examination to answer a specific psychiatric protocol question. This prompt enforces strict formatting guidelines, requiring the use of full, coherent sentences without bullet points or introductions, and demanding clear reasoning based on the provided evidence. Conversely, the Management Plan Evaluation Prompt (Supplementary Prompt 12) tasks a system with acting as a "psychiatric protocol response evaluator" to assess the degree to which a "Candidate Answer" matches a "Reference Answer". The evaluation ignores writing style to focus strictly on clinical correctness, protocol adherence, and the alignment of reasoning. The evaluator assigns a numerical score from 0 to 100 based on specific criteria, including protocol adherence, accuracy, completeness of Response, and Reasoning and Justification Alignment.

1.9 Clinical approach Task

The Clinical Approach Prediction Prompt (Supplementary Prompt 13) sets up a multi-stage task involving the generation and validation of clinical inquiries. It includes instructions to generate a single, focused, and medically relevant question derived from a specific "Clinical Approach" and patient history, as well as prompts for a psychiatrist persona to answer these questions appropriately. The framework also includes a grading component to verify if the generated questions contain keywords or semantic meanings directly related to the specified clinical approach. The corresponding Clinical Approach Evaluation Prompt (Supplementary Prompt 14) instructs a "mental health case response evaluator" to assess a "Candidate Answer" against a "Reference Answer" by focusing on clinical reasoning, assessment strategy, and diagnostic insight rather than writing style. The evaluator scores the response from 0 to 100 based on specific criteria: the accuracy and completeness of the assessment strategy, the validity of the diagnostic reasoning, the inclusion of necessary evaluation steps (such as screening tools or lab tests), and the alignment of the supporting rationale.

1.10 Mental QA Task

To assess the model's grasp of specific mental health concepts, a two-stage prompt pipeline was employed. The first prompt focused on concept prediction, explicitly directing the model to define the target mental-health concept clearly and concisely, mirroring the style of an exam review book to ensure conceptual precision and a professional tone. The second prompt was used for evaluation, instructing an automated scorer to assign a numeric accuracy score based on the completeness, clarity, depth, and relevance of the model's definition compared to the provided clinical history. The full wording of these prompts is provided in Mental QA Prediction prompt shown in Supplementary Prompt 15 .

1.11 Sequential Question Answering Task

To assess the model's capability in navigating complex, multi-step clinical reasoning, a sequential question answering framework was employed. The prediction prompt instructed the model to analyze patient history and address six distinct clinical domains: differential diagnosis, supporting historical evidence, etiological factors, treatment options, prognosis, and DSM-5-TR classification. To validate these outputs, the evaluation prompts guided an automated judge to compare the candidate's responses against expert reference answers, assigning numeric scores for each domain based on clinical accuracy and reasoning before computing a final average score. The full wording of these prompts is provided in Supplementary Prompt 16, Supplementary Prompt 17, and Supplementary Prompt 18.

1.12 Multiple-Choice Question Task

To evaluate the model's performance on objective assessment items, a structured multiple-choice question (MCQ) prompt was utilized. This prompt, designated as MCQ Prompt 1, directs the model to analyze the provided clinical history and logically assess the available options to select the best answer. Crucially, the instruction enforces a strict output format by requiring a single English letter as the response and explicitly prohibiting any accompanying explanation. This constraint ensures that the evaluation focuses solely on the accuracy of the final decision. The full wording of this prompt is provided in Supplementary Prompt 19

1.12.1 Separated Extended Matching Item

To rigorously assess the stability of model reasoning under varying presentation formats, the evaluation framework incorporated a Separated Extended Matching Item (EMI) task. This approach involved decomposing traditional, multi-question EMI sets into individual, standalone subquestions, thereby isolating specific clinical queries from the potential cross-item cues or interference present in the full-set format. The associated prompt structure presents the model with the necessary context—including the header, required condition, specific question, and choices—while enforcing a strict single-letter output format without explanation. This separation allows for a precise comparison between the model's performance on holistic case-based sets and its accuracy on atomic, context-independent items. Supplementary Prompt 20a

1.13 Classification Mapping

This subsection details the standardized diagnostic taxonomy employed for the classification task, which was explicitly derived from the DSM-5-TR to ensure terminological precision. To facilitate rigorous multi-label evaluation, two distinct reference lists were curated and validated by a licensed psychiatrist : a granular list of Specific Disorders Supplementary Prompt 21, encompassing detailed diagnoses such as "Bipolar I Disorder," and a high-level list of Disorder Categories Supplementary Prompt 22, which groups conditions into broader classes like "Depressive Disorders." These mappings serve as the ground truth for evaluation, requiring models to align their outputs with authoritative psychiatric nosology rather than relying on ambiguous or unstructured free-text descriptions

Supplementary Prompt 4. Diagnosis Prediction Prompt

> Analyze patient history. I will provide patient information and history. Use this information to answer the questions on the case.
>
> The Patient's History: {History}
>
> Question: {Question}

Supplementary Prompt 5. Diagnosis Evaluation Prompt

You are a medical diagnosis evaluator. Your task is to compare two medical text summaries and assess the degree to which the first text (the "Candidate Answer") captures the essential diagnostic information contained in the second text (the "Reference Answer"). Focus on the core medical meaning and the completeness of the diagnoses, *not on the writing style or specific wording.

User:
Instructions:
1. Read the "Reference Answer" carefully. Identify the key diagnoses (both primary and differential) and any crucial supporting information (e.g., symptoms, test results, ruled-out disorders).
2. Read the "Candidate Answer". The "Candidate Answer" will be a concise list of diagnoses and key findings.
3. Compare the "Candidate Answer" to the "Reference Answer" based on the following criteria:
Diagnostic Accuracy: Does the "Candidate Answer" correctly identify the most likely diagnosis (if one is clearly indicated in the "Reference Answer")?
Diagnostic Completeness: Does the "Candidate Answer" include *all of the major differential diagnoses mentioned in the "Reference Answer"? It is acceptable if the "Candidate Answer" uses slightly different terminology, as long as the underlying medical *concept is the same.
Supporting Information: Does the "Candidate Answer" include the key symptoms, test results, or other evidence that support the diagnoses?
It doesn't need to include *every detail, but it should capture the most important ones.
It's ok if this supporting information is implicitly present in the reference answer.
Exclusion of Irrelevant Information: Do *not penalize the "Candidate Answer" for including additional information that is not present in the "Reference Answer", *as long as that information is medically accurate and relevant to the case*.
Groundance does not affect the score: Do not penalize a response for being redundant.
4. Provide a single numerical similarity score between 0 and 100, where:
0: The "Candidate Answer" has no meaningful overlap with the "Reference Answer" in terms of diagnosis or supporting information. It's completely wrong.
50: The "Candidate Answer" identifies *some relevant diagnoses or supporting information, but it misses major elements or includes significant inaccuracies.
75: The "Candidate Answer" correctly identifies the primary diagnosis (if applicable) and most of the important differential diagnoses and supporting information. It may miss some minor details or include some slight inaccuracies.
90: The "Candidate Answer" is very close to the "Reference Answer" in terms of core diagnostic meaning and completeness. It may have minor omissions or differences in emphasis, but it captures almost all of the essential information.
100: The "Candidate Answer" is a perfect match in terms of core diagnostic meaning. It includes all of the key diagnoses and supporting information from the "Reference Answer", even if the wording or organization is different.
Do not justify or explain output only the numeric score.
5. Do not justify or explain the answer. Give a score between 0 and 100, no other text. Now, evaluate the following

Reference Answer: {Answer}

Candidate Response: {Response}

Supplementary Prompt 6. Treatment Prediction Prompt

```
You are a psychiatrist. Based on the patient's clinical history,answer the following question with
a medically appropriate and individualized treatment recommendation.
Guidelines
- Base your treatment recommendation primarily on the Patient's History. Use information from
the Mental and Physical Examinations only if it directly supports your treatment choice (if found).
- Your response should be clinically sound, concise, and focused on the patient's
specific presentation.
- Justify your recommendation using evidence from the case. Do not include general advice or
unrelated
commentary.
- Respond in full, professional sentences.

The Patient's History :{History}

Patient's Mental State Examination : {Mental State Examination}

Patient's Physical Examination : {Physical Examination}

Question : {Question}
```

Supplementary Prompt 7. **Treatment Evaluation Prompt**

```
SYSTEM: You are a medical treatment evaluator. Your task is to compare two medical treatment
summaries and assess the degree to which the first text (the "Candidate Answer") captures the
essential therapeutic interventions described in the second text (the "Reference Answer").
Focus on the medical appropriateness and completeness of the treatment plan, not on the writing
style or specific wording.
USER:
INSTRUCTIONS:
1. Read the "Reference Answer" carefully. Identify the key components of the treatment plan,
including primary therapeutic interventions, medications, behavioral strategies, or other relevant
clinical actions.
2. Read the "Candidate Answer". The "Candidate Answer" will be a concise summary of the proposed
treatments and strategies.
3. Compare the "Candidate Answer" to the "Reference Answer" based on the following criteria:
    Treatment Accuracy: Does the "Candidate Answer" correctly identify the key treatment or
    intervention as outlined in the "Reference Answer"? Treatment Completeness:
    Does the "Candidate Answer" include all of the major treatment components mentioned in the
    "Reference Answer"? Slightly different terminology is acceptable as long as the clinical
    *concept is preserved.Supporting Rationale: Does the "Candidate Answer" capture the reasoning or
    context for the treatment (e.g., why a specific method or medication is chosen)?
    It doesn't need to list every detail, but should reflect the main justification.Exclusion of
    Irrelevant Information: Do *not penalize the "Candidate Answer" for including additional,
    medically accurate and relevant information not found in the "Reference Answer".groundance does
    not affect the score: Do not penalize a response for being redundant.
4. Provide a single numerical similarity score between 0 and 100, where:
    0: The "Candidate Answer" has no meaningful overlap with the "Reference Answer" in terms
    of treatment or rationale. It is completely inappropriate or unrelated.
    50: The "Candidate Answer" identifies *some relevant treatment components or strategies, but
    misses key parts or includes significant inaccuracies.
    75: The "Candidate Answer" correctly identifies the primary treatment and most important
    additional strategies or reasoning. Minor omissions or slight inaccuracies are acceptable.
    90: The "Candidate Answer" is very close to the "Reference Answer" in terms of core treatment
    strategies and rationale. It may have minor differences in emphasis or detail.
    100: The "Candidate Answer" is a perfect match in terms of treatment content and rationale.
    It includes all key interventions and supporting reasoning from the "Reference Answer",
    even if the wording or structure is different.
5.Do not justify or explain the answer.Give a score between 0 and 100, no other text.Now evaluate
the following

Reference Answer:{Answer}
Candidate Answer:{Response}
```

Supplementary Prompt 8. Treatment Follow Up Prediction Prompt

As a psychiatrist, analyze the provided patient's information, history, and follow ups to answer the question according to the guidelines.
Guidelines
- Provide reasoning in your answer.
- Don't use bullet points just sentences.
- No introduction before the answer just provide the answer for the provided question only.
The Patient's History: {History}

```
if {First Follow Up}:
   follow = f"""First Follow Up {First Follow Up}"""
   prompt_template = prompt_template + follow
if {Second Follow up}:
   follow = f"""Second Follow up:{Second Follow up}"""
   prompt_template = prompt_template + follow
q = f"""Question: {Question}"""
prompt_template = prompt_template + q
```

Supplementary Prompt 9. Treatment Follow Up Evaluation Prompt

```
SYSTEM: You are a mental health case response evaluator. Your task is to compare two text responses
about a specific mental health case, assessing the degree to which the first text (the "Candidate
Answer") captures the essential information regarding the case assessment and recommended follow-up
contained in the second text (the "Reference Answer"). Focus on the core clinical meaning,
the completeness of the assessment understanding, and the appropriateness/completeness of the
follow-up plan, *not on writing style or specific wording.
User:
Instructions:
   1. Read the "Reference Answer" carefully. Identify the key aspects of the case assessment
   (e.g., presenting problem, relevant history, potential diagnoses/formulations, risk assessment)
   and the core elements of the recommended follow-up plan (e.g., therapy recommendations,
   medication considerations,
   further assessments needed, safety planning, referrals).
   2. Read the "Candidate Answer". This answer will also describe an assessment of the case and propose
   a follow-up plan.
   3. Compare the "Candidate Answer" to the "Reference Answer" based on the following criteria:
   Assessment Accuracy & Completeness: Does the "Candidate Answer" accurately reflect the core
   understanding of the patient's situation (presenting issues, key historical factors, potential
   clinical formulation/diagnosis) as presented in the "Reference Answer"? Does it capture the most
   critical elements of the assessment? Follow-up Plan Completeness & Appropriateness: Does the
   "Candidate Answer" include the essential elements and intent of the follow-up plan outlined in the
   "Reference Answer"? This includes key interventions (therapy, medication), monitoring, safety
   considerations, and necessary referrals.Minor variations in
   terminology are acceptable if the underlying clinical concept and action are the same. Rationale/
   Supporting Information: Does the "Candidate Answer" implicitly or explicitly capture the key
   reasons or clinical justifications for the assessment and follow-up plan that are present in the
   "Reference Answer"? It doesn't
   need every detail but should align on the core reasoning.Exclusion of Irrelevant Information:
   Do not penalize the "Candidate Answer" for including additional information *as long as that
   information is clinically accurate and relevant to the mental health case and its management*.
   However, significantly contradictory or inappropriate additions may lower the score. groundance
   does not affect the score: Do not penalize a response for being redundant.
   4. Provide a single numerical similarity score between 0 and 100, where:
      0: The "Candidate Answer" has no meaningful overlap with the "Reference Answer" in terms of case
      assessment or follow-up plan. It's completely different or clinically inappropriate.
      50: The "Candidate Answer" identifies some relevant aspects of the assessment or follow-up plan
      but misses major elements, contains significant inaccuracies, or proposes a substantially
      different approach.
      75: The "Candidate Answer" correctly captures the main aspects of the assessment and most of the
      essential follow-up recommendations from the "Reference Answer". It may miss some minor details,
      nuances, or justifications.
      90: The "Candidate Answer" is very close to the "Reference Answer" in terms of core case
      understanding and the proposed follow-up plan. It may have minor omissions or differences in
      emphasis but captures almost all essential clinical information and actions.
      100: The "Candidate Answer" perfectly matches the "Reference Answer" in terms of core clinical
      meaning regarding the assessment and follow-up plan. It includes all key assessment points and
      recommended actions, even if the wording or organization differs. Do not justify or explain the
      answer. Give a score between 0 and 100, no other text.Now, evaluate the following
Reference Answer: {Answer}
Candidate Response: {Response}
```

Supplementary Prompt 10. Classification Prompt

```
As a psychiatrist, analyze the provided patient's information and history to determine if
the patient
exhibits clear symptoms of any of the provided list of disorders according to provided guidelines.
Guidelines
- The patient could have multiple illness at the same time else he is normal.
- Concise Response: Respond with any combination of these disorders separated by a comma
and if the poster doesn't have any mental illness just answer with Normal.
- No Explanations: Don't providing explanations for your assessment.
- Ambiguity: If the it is unclear, choose the most probable label/disorder.
Disorders List:
The Disorders List (Categories or Specific Disorders)
The Patient's History:
{History}
```

Supplementary Prompt 11. Management Plan Prediction Prompt

```
You are a psychiatrist. Analyze the provided patient's information(History, Mental State Examination,
and Physical Examination) to answer the psychiatric protocol question according to the guidelines
below. Guidelines:
- Provide clear reasoning in your answer based on the information provided.
- Use full, coherent sentences only; do not use bullet points.
- Do not include an introduction or any prefatory remarks; start directly with the answer.
The Patient's History:
{History}
Patient's Mental State Examination:
{Mental State Examination}
Patient's Physical Examination:
{Physical Examination}
Question:
{Question}
```

Supplementary Prompt 12. Management Plan Evaluation Prompt

```
SYSTEM: You are a psychiatric protocol response evaluator. Your task is to compare two text responses
about a psychiatric protocol
question, assessing the degree to which the first text (the "Candidate
Answer") captures the essential information contained in the second text (the "Reference Answer" or
"True Label"). Focus on the clinical correctness, completeness of protocol\adherence, and the
accuracy of reasoning, *not on writing style or exact wording.
User:
Instructions:
1. Read the "Reference Answer" carefully. Identify the key components expected in the psychiatric
protocol response (e.g., symptom recognition, diagnostic steps, risk/safety considerations,
treatment recommendations ethical/legal procedures if applicable).
2. Read the "Candidate Answer". This answer will attempt to respond to the same psychiatric protocol
question.
3. Compare the "Candidate Answer" to the "Reference Answer" based on the
following criteria:
    Protocol Adherence & Accuracy: Does the "Candidate Answer" correctly follow the
    psychiatric protocol steps presented in the "Reference Answer"? Does it recognize key clinical
    issues, decision points, and require actions?  Completeness of Response: Does the
"Candidate Answer" address all major elements expected by the protocol as reflected in the
"Reference Answer" (e.g., diagnostic considerations, intervention steps, risk management)?
Reasoning and Justification Alignment: Does the "Candidate Answer" capture the rationale
    (explicit or implied) behind the steps taken according to the protocol in the  "Reference Answer"?
    It should reflect the clinical reasoning that justifies  assessment and management  decisions.
    Handling of Additional Information: Extra information is acceptable if it is clinically correct
    and protocol-consistent. Do not penalize additional relevant detail. However, incorrect,
    contradictory, or unsafe suggestions should lower the score. groundance Tolerance: groundance
    (repeating points) does not affect the score.

4. Provide a single numerical similarity score between 0 and 100, where:
    0: The "Candidate Answer" has no meaningful overlap with the "Reference Answer" in terms of
    protocol steps,clinical reasoning, or appropriate action.
    50: The "Candidate Answer" captures some relevant elements but misses major steps, has serious
    inaccuracies or suggests significantly incorrect approaches.
    75: The "Candidate Answer" captures most key protocol elements and clinical reasoning, with
    only minor omissions or inaccuracies.
    90: The "Candidate Answer" is very close to the "Reference Answer" in terms of psychiatric protocol
    adherence, clinical assessment, and recommended actions,  with only slight gaps or minor emphasis
    differences.
    100: The "Candidate Answer" fully matches the "Reference Answer" in terms of psychiatric
    protocol steps, reasoning, and recommended actions, even if wording or order differ.

5. Do not justify or explain the answer. Only provide a single numerical score between 0 and 100.
No other text output.Now, evaluate the following
Reference Answer: {Answer}
Candidate Response: {Response}
```

Supplementary Prompt 13. Clinical approach Prompt

```
The Patient's History: {History}
Clinical Approach: {Clinical Approach}
Generated Question: Generate exactly one clear, focused, and medically relevant question based on
Clinical Approach.
```

Clinical approach Question Generation Prompt

```
You are an expert grader evaluating whether a history question is relevant to a given clinical
approach. Question: {Question_Generation}
Clinical Approach: {Clinical Approach}
Determine if the question contains keywords or semantic meaning that are directly related to
the clinical approach. Respond only with "yes" if it is relevant or "no" if it is not.
Do not provide any explanation.
```

Clinical approach Grading Prompt

```
You are a psychiatrist. Based on the patient's clinical history and the question, provide a clear,
accurate, and medically appropriate answer.
The Patient's History:{History}
Question: {Question_Generation}
```

Clinical approach Answer Generation Prompt

Supplementary Prompt 14. Clinical Approach Evaluation Prompt

```
SYSTEM: You are a mental health case response evaluator. Your task is to compare two text responses
about a specific mental health case, assessing the degree to which the first text (the
"Candidate Answer") captures The essential information regarding the clinical approach is
contained in the second text("Reference Answer") Focus on the clinical reasoning, assessment
strategy, and diagnostic insight, not on writing style .
User:
Instructions:
1. Read the "Reference Answer" carefully. Identify the key components of the clinical approach:
presenting symptoms, history taking, differential diagnosis, initial evaluation steps, and rationale
for assessment strategy.
2. Read the "Candidate Answer". This is another attempt to outline a clinical approach to the same case
3. Compare the "Candidate Answer" to the "Reference Answer" based on the following criteria:
   Assessment Strategy Accuracy & Completeness: Does the "Candidate Answer" reflect an accurate and
   complete approach toassessing the case? Does it capture the key symptoms, relevant history,
   and potential clinical hypotheses found in the "Reference Answer"? Diagnostic Reasoning &
   Thought Process: Does the "Candidate Answer" follow a similar line of clinical reasoning,
   including formulation or differential diagnosis as in the "Reference Answer"? Evaluation Steps:
   Does it propose similar or equivalent steps in the evaluation process (e.g., screeningtools,
   physical exams, lab tests, psychiatric interview elements)?
   Rationale Alignment: Does the "Candidate Answer" align with the clinical logic and
   justifications provided in the "Reference Answer"? Perfect wording is not needed focus on
   reasoning.No Penalty for Relevance: Do not penalize clinically valid additions if they are
   appropriate, even if not found
   in the"Reference Answer". groundance does not grace score.
4. Provide a single numeric score between 0 and 100:
   0: Completely off-topic, unrelated, or clinically incorrect.
   50:Some valid elements of clinical approach are present, but many key elements are missing or
   poorly reasoned.
   75: Mostly aligned with correct steps and rationale, missing only some secondary elements.
   90: Nearly identical clinical reasoning and plan; may differ in phrasing.
   100: Fully matches the core clinical approach and rationale.
   Do not justify or explain output only the numeric score. Now, evaluate the following
Reference Answer: {Clinical approach}
Candidate Response: {Response}
```

Supplementary Prompt 15. Two-stage Mental QA prompt pipeline. (a) Prediction Prompt directs the model to define the target mental-health concept clearly and concisely, as in an exam-style review, emphasizing conceptual precision and professional tone; (b) Evaluation Prompt instructs an automated scorer to assign a 0–100 accuracy score based on the completeness, clarity, depth, and relevance of the model's definition.

```
You are a psychiatrist specializing in mental health knowledge. Based on the provided dataset,
answer the following question accurately and concisely. Ensure your response is well-structured,
using bullet points or number lists where appropriate, and maintain a professional tone throughout.
Think step by step to provide a logical and coherent answer like those found in exam review books.
The Patient's History: {History}
Question: {Question}
```

Mental QA Prediction Prompt

```
System: You are a mental health concept knowledge evaluator. Your task is to assess how accurately,
completely, and clearly, the candidate's response defines the concept provided in the "Answer" field,
taking into account the clinical context in the "History."
User:
Instructions:
   1. Read the "Answer" this is the clinical concept or term to define
   (e.g., "Loss of interest or pleasure in activities").
   2. Read the "Candidate Response" the model's definition/explanation of that concept.
   3. Evaluate the response on:Definition Accuracy & Completeness: Are all core features of the
   concept present and correctly described? Clarity & Precision:Is the explanation clear,
   unambiguous, and clinically precise?Depth of Explanation: Does it include relevant examples or
   elaborations that demonstrate understanding?
   Relevance & Focus:Does it avoid irrelevant details and stick to the concept at hand?
   4. Provide a single numeric score between 0 and 100:
   0: No meaningful overlap incorrect or missing core elements.
   50: Some correct elements but major omissions or inaccuracies.
   75: Mostly correct with only minor gaps or imprecisions.
   90:Very close to a perfect definition; only small details missing.
   100:Perfectly accurate, complete, and clear.
   Do not justify or explain output only the numeric score.
Now, evaluate the following
Reference Answer : {Answer}
Candidate Response: {Response}
```

Mental QA Evaluation Prompt

Supplementary Prompt 16. Sequential Question Answering Prediction Prompt

```
The Patient's History: {History}
Task: Based on the provided history, answer the following Six clinical questions. Use the same
format and level of detail shown in the example. Make sure to include preferred and alternative
diagnoses where applicable, and support your answers with reasoning drawn fromthe case.
Q1: What is the likely differential diagnosis?
   preferred diagnosis:
   Alternative diagnoses:
Q2: What information in the history supports the diagnosis, and what other information
would help to confirm it?
   (Provide a detailed rationale using history details and explain what additional
   information/tests are needed to confirm the diagnosis.)
Q3 : What might the important etiological factors be?
   (Organize into: Pregrazing, Precipitating, Maintaining factors)
Q4: What treatment options are available?
   (Cover location-based options, physical treatments, psychological support,
   and social considerations. Mention use of Mental Health Act if relevant.)
Q5: What is the prognosis in this case?
   (Discuss short-term and long-term outlook. Include good and poor prognostic
   indicators based on patient specifics.)
Q6: based on the provided History ,which class does this diagnosis belong to according
to DSM-5-TR ?
```

Supplementary Prompt 17. Sequential Question Answering Evaluation Prompt (Part 1)

```
SYSTEM: You are a mental health case response evaluator. Your task is to assess six clinical responses
from a trainee clinician. Each response corresponds to a specific psychiatric question.
For each question, compare the candidate's answer to the expert reference answer and provide a numeric
score based on how well the candidate captures the clinical reasoning and content.

Q1: What is the likely differential diagnosis?
- Task Type: Diagnosis
- Focus: Diagnostic reasoning and clinical hypotheses.
- Scoring System:
   0: Completely off-topic, unrelated, or clinically incorrect.
   50: Some valid elements of the diagnosis are present, but many key elements are missing
   or poorly reasoned.
   75: Mostly aligned with correct steps and rationale, missing only some secondary elements.
   90: Nearly identical clinical reasoning and plan; may differ in phrasing.
   100: Fully matches the core diagnosis and rationale.
Concept to Define: {Answer for Question 1}
Candidate Response: {Response for Question 1}

Q2: What information in the history supports the diagnosis, and what other information would
help to confirm it?
- Task Type: History
- Focus: Presenting symptoms, clinical history, and psychosocial context.
- Scoring System:
   0: Completely off-topic, unrelated, or clinically incorrect.
   50: Some valid elements of the history are present, but many key elements are missing
   or poorly reasoned.
   75: Mostly aligned with correct steps and rationale, missing only some secondary elements.
   90: Nearly identical clinical reasoning and plan; may differ in phrasing.
   100: Fully matches the core history and rationale.
Concept to Define: {Answer for Question 2}
Candidate Response: {Response for Question 2}

Q3: What might the important aetiological factors be?
- Task Type: Aetiology
- Focus: Biological, psychological, and social contributing factors.
- Scoring System:
   0: Completely off-topic, unrelated, or clinically incorrect.
   50: Some valid elements of the aetiology are present, but many key elements are missing
   or poorly reasoned.
   75: Mostly aligned with correct steps and rationale, missing only some secondary elements.
   90: Nearly identical clinical reasoning and plan; may differ in phrasing.
   100: Fully matches the core aetiology and rationale.
Concept to Define: {Answer for Question 3}
Candidate Response: {Response for Question 3}

Q4: What treatment options are available?
- Task Type: Treatment
- Focus: Clinical interventions including medication, therapy, and lifestyle recommendations.
- Scoring System:
   0: Completely off-topic, unrelated, or clinically incorrect.
   50: Some valid elements of the treatment are present, but many key elements are missing or
   poorly reasoned.
   75: Mostly aligned with correct steps and rationale, missing only some secondary elements.
   90: Nearly identical clinical reasoning and plan; may differ in phrasing.
   100: Fully matches the core treatment and rationale.
Concept to Define: {Answer for Question 4}
Candidate Response: {Response for Question 4}
```

Supplementary Prompt 18. Sequential Question Answering Evaluation Prompt (Part 2)

```
Q5: What is the prognosis in this case?
- Task Type: Prognosis
- Focus: Outcome prediction, expected recovery, and risk factors.
- Scoring System:
   0: Completely off-topic, unrelated, or clinically incorrect.
   50: Some valid elements of the prognosis are present, but many key elements are missing
   or poorly reasoned.
   75: Mostly aligned with correct steps and rationale, missing only some secondary elements.
   90: Nearly identical clinical reasoning and plan; may differ in phrasing.
   100: Fully matches the core prognosis and rationale.
Concept to Define: {Answer for Question 5}
Candidate Response: {Response for Question 5}

Q6: Based on the provided history, which class does this diagnosis belong to according to DSM-5 or
ICD-10?
- Task Type: Classification
- Focus: Diagnostic nosology and classification system alignment.
- Scoring System:
   0: Completely off-topic, unrelated, or clinically incorrect.
   50: Some valid elements of the classification are present, but many key elements are missing
   or poorly reasoned.
   75: Mostly aligned with correct steps and rationale, missing only some secondary elements.
   90: Nearly identical clinical reasoning and plan; may differ in phrasing.
   100: Fully matches the core classification and rationale.
Concept to Define: {Answer for Question 6}
Candidate Response: {Response for Question 6}


Now:
- Return your scores only in this JSON format, with numeric values only.
- Then, sum the six scores and divide by 6 to compute the final score.
- Return only the final score as a single number (no labels, no JSON, no extra text).

{
 "Q1": ___,
 "Q2": ___,
 "Q3": ___,
 "Q4": ___,
 "Q5": ___,
 "Q6": ___
}
Final Score: ___
```

Supplementary Prompt 19. Prompting strategies for objective assessment items. (a) MCQ Prompt 1 directs the model to choose one answer without explanation; (b) EMI Prompt 2 blends scenario-based extended matching items (EMIs) with standard MCQs, requiring only the answer letters.

```
Answer the question using one of the given choices.Do not provide any explanation for your choice.
Indicate your choice with a single English letter.Provide only one answer;
Multiple answers could be provided the Patient's History:{History}
Question:{Question}
Choices:{Choices}
```

MCQ Prompt 1

```
Analyze the multiple-choice question (MCQ) and select the best answer.Assess each option logically
based on relevant psychological history.Do not provide any explanation for your choice. Indicate your
choice with a single English letter. (Provide only one answer; Multiple answers could be provided.)
The Patient's History:{History}
Question:{Question}
Choices:{Choices}
```

EMI Prompt 1

Supplementary Prompt 20. Point-based similarity EMI prompting templates: (a) a straightforward "header-required" template that simply presents the fields.

```
Answer the question using one of the given choices.Do not provide any explanation for your choice.
Indicate your choice with a single English letter. (Provide only one answer; Multiple answers could
be provided.)
Header:{Header}
Required:{Required}
Question:{Question}
Choices:{Choices}
```

(a) EMI Separated Prompt 1

Supplementary Prompt 21. Specific Disorders List

```
[ "Intellectual Developmental Disorder (Intellectual disability)",
"Global Developmental Delay", "Unspecified Intellectual Developmental Disorder",
"Language Disorder", "Speech Sound Disorder",
"Childhood-Onset Fluency Disorder (Stuttering)",
"Social (Pragmatic) Communication Disorder", "Unspecified Communication Disorder",
"Autism Spectrum Disorder", "Attention-Deficit/Hyperactivity Disorder",
"Other Specified Attention-Deficit/Hyperactivity Disorder",
"Unspecified Attention-Deficit/Hyperactivity Disorder","Specific Learning Disorder",
"Developmental Coordination Disorder", "Stereotypic Movement Disorder",
"Substance-Induced Repetitive Behaviors", "Tourette's Disorder",
"Persistent (Chronic) Motor or Vocal Tic Disorder", "Provisional Tic Disorder",
"Other specified Tic disorder", "Unspecified Tic Disorder",
"Other specified Neurodevelopmental disorders",
"Unspecified neurodevelopmental disorders","Delusional Disorder",
"Brief Psychotic Disorder",
"Schizophreniform Disorder", "Schizophrenia", "Schizoaffective Disorder",
"Substance/Medication-Induced Psychotic Disorder",
"Psychotic disorder due to another medical disorders",
"Catatonia associated with another mental disorder",
"Catatonia associated with another medical disorder", "Unspecified Catatonia",
"Other Specified Schizophrenia Spectrum and Other Psychotic Disorder",
"Unspecified Schizophrenia Spectrum and Other Psychotic Disorder", "Bipolar I Disorder",
"Bipolar II Disorder", "Cyclothymic Disorder",
"Substance/Medication-Induced Bipolar and Related Disorder",
"Bipolar and Related Disorder Due to Another Medical disorders",
"Other Specified Bipolar and Related Disorder",
"Unspecified Bipolar and Related Disorder",
"Unspecified Mood Disorder", "Disruptive Mood Dysregulation Disorder",
"Major Depressive Disorder", "Persistent Depressive Disorder",
"Premenstrual Dysphoric Disorder", "Substance/Medication-Induced Depressive Disorder",
"Depressive Disorder Due to Another Medical disorders",
"Other Specified Depressive Disorder", "Unspecified Depressive Disorder",
"Separation Anxiety Disorder", "Selective Mutism", "Specific Phobia",
"Social Anxiety Disorder",
"Panic Disorder", "Agoraphobia","Generalized Anxiety Disorder",
"Substance/Medication-Induced Anxiety Disorder",
"Anxiety Disorder Due to Another Medical disorders", "Other Specified Anxiety Disorder",
"Unspecified Anxiety Disorder", "Obsessive-Compulsive Disorder",
"Body Dysmorphic Disorder",
"Hoarding Disorder","Trichotillomania (Hair-Pulling Disorder)",
"Excoriation (Skin-Picking) Disorder",
"Substance/Medication-Induced Obsessive-Compulsive and Related Disorder",
"Obsessive-Compulsive and Related Disorder Due to Another Medical disorders",
"Other Specified Obsessive-Compulsive and Related Disorder",
"Unspecified Obsessive-Compulsive and Related Disorder", "Reactive Attachment Disorder",
"Disinhibited Social Engagement Disorder", "Posttraumatic Stress Disorder",
"Acute Stress Disorder", "Adjustment Disorders", "Prolonged Grief Disorder",
"Other Specified Trauma- and Stressor-Related Disorder",
"Unspecified Trauma- and Stressor-Related Disorder", "Dissociative Identity Disorder",
"Dissociative Amnesia", "Depersonalization/Derealization Disorder",
"Other Specified Dissociative Disorder", "Unspecified Dissociative Disorder",
"Somatic Symptom Disorder", "Illness Anxiety Disorder",
"Functional Neurological Symptom Disorder (Conversion Disorder)",
"Psychological Factors Affecting Other Medical disorders", "Factitious Disorder",
"Other Specified Somatic Symptom and Related Disorders",
"Unspecified Somatic Symptom and Related Disorder", "Pica", "Rumination Disorder",
```

```
"Avoidant/Restrictive Food Intake Disorder", "Anorexia Nervosa", "Bulimia Nervosa",
"Binge-eating disorder", "Other Specified Feeding or eating disorder",
"Unspecified Feeding or eating disorder", "Enuresis", "Encopresis",
"Other Specified Elimination Disorder", "Unspecified Elimination Disorder",
"Insomnia Disorder", "Hypersomnolence Disorder", "Narcolepsy",
"Obstructive Sleep Apnea Hypopnea", "Central Sleep Apnea",
"Sleep-Related Hypoventilation", "Delayed Sleep Phase Type",
"Advanced Sleep Phase Type", "Irregular Sleep-Wake Type",
"Non-24-Hour Sleep-Wake Type", "Shift Work Type", "Parasomnias",
"Non-Rapid Eye Movement Sleep Arousal Disorders", "Nightmare Disorder",
"Rapid Eye Movement Sleep Behavior Disorder", "Restless Legs Syndrome",
"Substance/Medication-Induced Sleep Disorder", "Other Specified Insomnia Disorder",
"Unspecified Insomnia Disorder", "Other Specified Hypersomnolence Disorder",
"Unspecified Hypersomnolence Disorder", "Other Specified Sleep-Wake Disorder",
"Unspecified Sleep-Wake Disorder", "Delayed Ejaculation", "Erectile Disorder",
"Female Orgasmic Disorder", "Female Sexual Interest/Arousal Disorder",
"Genito-Pelvic Pain/Penetration Disorder", "Male Hypoactive Sexual Desire Disorder",
"Premature (Early) Ejaculation", "Substance/Medication-Induced Sexual Dysfunction",
"Other Specified Sexual Dysfunction", "Unspecified Sexual Dysfunction",
"Other Specified Gender Dysphoria", "Unspecified Gender Dysphoria",
"Oppositional Defiant Disorder","Intermittent Explosive Disorder","Conduct Disorder",
"Pyromania", "Kleptomania",
"Other Specified Disruptive, Impulse-Control, and Conduct Disorder",
"Unspecified Disruptive, Impulse-Control, and Conduct Disorder",
"Alcohol Use Disorder", "Alcohol Intoxication", "Alcohol Withdrawal",
"Alcohol-induced Mental disorders", "Unspecified Alcohol-Related Disorder",
"Caffeine Intoxication", "Caffeine Withdrawal", "Caffeine-Induced Mental Disorders",
"Unspecified Caffeine-Related Disorder", "Cannabis Use Disorder",
"Cannabis Intoxication", "Cannabis Withdrawal", "Cannabis-Induced Mental Disorders",
"Unspecified Cannabis-Related Disorder", "Phencyclidine Use Disorder",
"Other Hallucinogen Use Disorder", "Phencyclidine Intoxication",
"Other Hallucinogen Intoxication", "Hallucinogen Persisting Perception Disorder",
"Phencyclidine-Induced Mental Disorders", "Hallucinogen-Induced Mental Disorders",
"Unspecified Phencyclidine-Related Disorder",
"Unspecified Hallucinogen-Related Disorder", "Inhalant Use Disorder",
"Inhalant Intoxication", "Inhalant-Induced Mental Disorders",
"Unspecified Inhalant-Related Disorder","Opioid Use Disorder","Opioid Intoxication",
"Opioid Withdrawal", "Opioid-Induced Mental Disorders",
"Unspecified Opioid-Related Disorder",
"Sedative, Hypnotic, or Anxiolytic Use Disorder",
"Sedative, Hypnotic, or Anxiolytic Intoxication",
"Sedative, Hypnotic, or Anxiolytic Withdrawal",
"Sedative-, Hypnotic-, or Anxiolytic-Induced Mental Disorders",
"Unspecified Sedative-, Hypnotic-, or Anxiolytic- ]
```

Supplementary Prompt 22. Disorder Categories List

```
["Intellectual Developmental Disorders", "Communication Disorders", "Autism Spectrum Disorder",
"Attention-Deficit/Hyperactivity Disorder", "Specific Learning Disorder", "Motor Disorders",
"Tic Disorders","Schizophrenia Spectrum and Other Psychotic Disorders", "Bipolar and Related Disorders",
"Depressive Disorders", "Anxiety Disorders","Obsessive-Compulsive and Related Disorders",
"Trauma- and Stressor-Related Disorders", "Dissociative Disorders", "Somatic Symptom and Related Disorders",
"Feeding and Eating Disorders", "Elimination Disorders", "Sleep-Wake Disorders", "Parasomnias",
"Sexual Dysfunctions","Breathing-Related Sleep Disorders", "Gender Dysphoria", "Disruptive,
Impulse-Control, and Conduct Disorders", "Substance-Related and Addictive Disorders",
"Neurocognitive Disorders", "Personality Disorders", "Medication-Induced Movement Disorders",
"Paraphilic Disorders"]
```
